# Prompt Reduction in CRP, IL-6, IFN-γ, IP-10, and MCP-1 and a Relatively Low Basal Ratio of Ferritin/CRP Is Possibly Associated With the Efficacy of Tocilizumab Monotherapy in Severely to Critically Ill Patients With COVID-19

**DOI:** 10.3389/fmed.2021.734838

**Published:** 2021-09-23

**Authors:** Shoji Hashimoto, Kazuyuki Yoshizaki, Kazuko Uno, Heita Kitajima, Tsuyoshi Arai, Yoshitaka Tamura, Hiroshi Morishita, Hiroto Matsuoka, Yuki Han, Seijiro Minamoto, Tomonori Hirashima, Tomoki Yamada, Yozo Kashiwa, Makoto Kameda, Seiji Yamaguchi, Yasunari Tsuchihashi, Mitsuhiro Iwahashi, Emi Nakayama, Tatsuo Shioda, Takayuki Nagai, Toshio Tanaka

**Affiliations:** ^1^Osaka Prefectural Hospital Organization Osaka Habikino Medical Center, Osaka, Japan; ^2^Department of Organic Fine Chemicals, Institute of Scientific and Industry Research, Osaka University, Osaka, Japan; ^3^Division of Basic Research, Louis Pasteur Center for Medical Research, Kyoto, Japan; ^4^Higashi Hiroshima Memorial Hospital, Hiroshima, Japan; ^5^Department of Viral Infection, Research Institute for Microbial Diseases, Osaka University, Osaka, Japan

**Keywords:** COVID-19, cytokine storm, IL-6, tocilizumab, dexamethasone

## Abstract

**Background and Aim:** Tocilizumab, a humanized anti-IL-6 receptor antibody, has been used to treat severely to critically ill patients with COVID-19. A living systematic review with meta-analysis of recent RCTs indicates that the combination therapy of corticosteroids and tocilizumab produce better outcomes, while previous observational studies suggest that tocilizumab monotherapy is beneficial for substantial numbers of patients. However, what patients could respond to tocilizumab monotherapy remained unknown.

**Methods:** In this retrospective study we evaluated the effects of tocilizumab monotherapy on the clinical characteristics, serum biomediator levels, viral elimination, and specific IgG antibody induction in 13 severely to critically ill patients and compared with those of dexamethasone monotherapy and dexamethasone plus tocilizumab.

**Results:** A single tocilizumab administration led to a rapid improvement in clinical characteristics, inflammatory findings, and oxygen supply in 7 of 11 patients with severe COVID-19, and could recover from mechanical ventilation management (MVM) in 2 patients with critically ill COVID-19. Four patients exhibited rapidly worsening even after tocilizumab administration and required MVM and additional methylprednisolone treatment. Tocilizumab did not delay viral elimination or inhibit IgG production specific for the virus, whereas dexamethasone inhibited IgG induction. A multiplex cytokine array system revealed a significant increase in the serum expression of 54 out of 80 biomediators in patients with COVID-19 compared with that in healthy controls. Compared with those who promptly recovered in response to tocilizumab, patients requiring MVM showed a significantly higher ratio of basal level of ferritin/CRP and a persistent increase in the levels of CRP and specific cytokines and chemokines including IL-6, IFN-γ, IP-10, and MCP-1. The basal high ratio of ferritin/CRP was also associated with clinical deterioration even in patients treated with dexamethasone and tocilizumab.

**Conclusion:** Tocilizumab as monotherapy has substantial beneficial effects in some patients with severe COVID-19, who showed a relatively low level of the ratio of ferritin/CRP and prompt reduction in CRP, IL-6, IFN-γ, IP-10, and MCP-1. The high ratio of ferritin/CRP is associated with rapid worsening of pneumonia. Further evaluation is warranted to clarify whether tocilizumab monotherapy or its combination with corticosteroid is preferred for severely to critically ill patients with COVID-19.

## Introduction

Coronavirus disease 2019 (COVID-19), caused by severe acute respiratory syndrome coronavirus-2 (SARS-CoV-2), has rapidly spread worldwide (1, 2). By the middle of May in 2021, over 160 million people had been diagnosed with COVID-19 with a mortality rate of ~2.1 percentage. Thus, vaccines and therapeutic agents are urgently needed to stop the spread of this disease and reduce the associated mortality; however, a few vaccines and no drugs except for remdesivir, dexamethasone, and baricitinib have been developed and approved (3–5). A cytokine storm, also known as hyperinflammation, is the pathological mechanism underlying the development of severe disease, leading to a critically ill state of patients, which is characterized by acute respiratory distress syndrome, multiple organ dysfunction, and shock (6, 7). Among biomediators involved in severe and critical cases of COVID-19, interleukin (IL)-6 is highly elevated and can be used a prognostic marker (8, 9). Additionally, IL-6 plays a major pathological role in disease worsening (10).

Thus far, recent various randomized, controlled trials (RCTs) of tocilizumab, a humanized anti-IL-6 receptor monoclonal antibody, for severe and critical COVID-19 showed mixed results (11, 12), while large scale of RCTs such as REMAP-CAP and RECOVERY supported the efficacy of tocilizumab in severe to critical COVID19 when treated in combination with corticosteroids (13, 14). However, there are concerns that the combination therapy inhibits viral elimination and induces various adverse events (15). In contrast, several observational studies reported the beneficial effects of tocilizumab monotherapy in patients with COVID-19 (16–19). However, what patients with severe COVID-19 could respond to tocilizumab monotherapy remained to be determined.

Based on previous clinical and laboratory findings of COVID-19 (1, 8, 9, 16), our hospital used tocilizumab to treat severely to critically ill patients with COVID-19 until the end of June in 2020. After August in 2020 dexamethasone was routinely used for patients requiring oxygen support if needed in combination with tocilizumab and we retrospectively analyzed their effects on the clinical characteristics, viral clearance, IgG antibody induction against SARS-CoV-2, and levels of multiple biomediators including cytokines, chemokines, and soluble receptors.

## Methods

### Patients With COVID-19

By the end of June in 2020, 13 patients with COVID-19 admitted to our hospital were diagnosed as being severely to critically ill, as they required oxygen supply because of severe pneumonia and were intravenously administered tocilizumab at 400 mg once in combination with potential anti-SARS-CoV-2 drugs such as lopinavir/ritonavir, ciclesonide, or favipiravir. In July dexamethasone proved to be effective for reducing mortality of severely to critically ill patients with COVID-19 (4), thereafter dexamethasone was routinely used for the treatment of severely ill COVID-19, while tocilizumab was subsequently administered for further worsening patients. All patients provided written informed consent and the off-label compassionate use of tocilizumab was approved by the Ethics Committee of Osaka Habikino Medical Center (Approved ID: 150-7).

### Patients With Influenza (2009H1N1), Rheumatoid Arthritis, and Idiopathic Multicentric Castleman Disease

To compare the expression profile of cytokines and chemokines in patients with COVID-19, nine patients with influenza (2009H1N1), 28 patients with rheumatoid arthritis (RA), and 19 patients with idiopathic multicentric Castleman disease (iMCD) were enrolled. Influenza virus infection was diagnosed using a rapid influenza antigen diagnostic test. The diagnosis of RA and iMCD was based on the American Rheumatism Association 1987 revised criteria for the classification of RA and the international, evidence-based diagnostic criteria for HHV-8-negative/iMCD, respectively (20, 21). Ethical approval was obtained from the Higashi Hiroshima Memorial Hospital Ethical Committee (approval number: HMH-09-03) for RA, from the Ethics Committee of Tokushukai Hospital (approval number: TGEO-1391-014, TGEO-1391-071, and TGEO-1547-004) for iMCD, and from the Ethics Committee of Louis Pasteur Center for Medical Research for influenza and healthy subjects (approval number: LPC.8). All participants provided their written informed consent.

### Our Guidance for the Indication of Tocilizumab for Patients With COVID-19

Based on various previous reports (1, 8, 9, 16), the inclusion criteria of the off-label indication of tocilizumab were set as follows:

1. Elevated inflammatory findings: C-reactive protein (CRP) level >5 mg/dL or ferritin level >1,000 ng/mL
2. Requirement of oxygen supply or rapid progression according to chest radiographic evaluation (more than 50% increase in infiltrates over 24–48 h).

Patients with elevated procalcitonin and those with concomitant bacterial infections were excluded.

This guidance was kept on until the beginning of August when the reducing efficacy of dexamethasone on mortality of severely to critically ill patients with COVID-19 was verified. The indication of dexamethasone was requirement of oxygen supply, so for patients with severe COVID-19, regardless of inflammatory laboratory results.

### Measurement of Biomediators

The basal serum levels of cytokines, chemokines, and soluble receptors were quantified using Bio-Plex 200 (Bio-Rad Laboratories, CA, USA), a multiplex cytokine array system, according to the manufacturer's instructions. Briefly, blood sera from patients with severe to critical COVID-19 (*n* = 12), patients with influenza (*n* = 9), patients with RA (*n* = 28), and patients with iMCD (*n* =19) were collected and centrifuged at 1,600 × g for 10 min. Since age distribution was different among the patient group (Mean ± SD, COVID-19, 63.5 ± 4.2; influenza, 21.6 ± 1.81; RA, 61.1 ± 2.0; iMCD, 44.2 ± 2.9), age-matched healthy subjects, who presented no active acute inflammatory disease at the time of blood collection and exhibited no history of cancer, chronic infectious diseases, autoimmune diseases, nephritis, or asthma, were selected as controls for COVID-19 (*n* = 38, Mean ± SD, 59.4 ± 2.5), influenza (*n* = 10, Mean ± SD, 26.3 ± 1.8), RA and iMCD (*n* = 41, Mean ± SD, 52.9 ± 1.9). The serum samples were frozen at −80°C until further analysis. We simultaneously quantified 80 types of cytokines, chemokines, soluble receptors, and other mediators. The Bio-Plex Human Cytokine 27-Plex Panel included interleukin (IL)-1β, IL-1 receptor antagonist (IL-Ra), IL-2, IL-4, IL-5, IL-6, IL-7, IL-8, IL-9, IL-10, IL-12(p70), IL-13, IL-15, IL-17, basic fibroblast growth factor (bFGF), eotaxin, granulocyte colony stimulating factor (G-CSF), granulocyte macrophage CSF (GM-CSF), interferon (IFN) -γ, IFN-inducible protein of 10 kD (IP-10), monocyte chemoattractant protein-1 (MCP-1), macrophage inflammatory protein-1α (MIP-1α), MIP-1β, platelet-derived growth factor-BB (PDGF-BB), regulated on activation, normal T cell expressed and secreted (RANTES), tumor necrosis factor (TNF)-α, and vascular endothelial grow factor (VEGF). The Inflammation Panel Kit (Bio-Rad Laboratories, CA, USA) included a proliferation-inducing ligand (APRIL), B cell-activating factor belonging to the TNF family (BAFF), soluble (s)CD30, sCD163, chitinase, sgp130, IFN-α2, IFN-β, sIL-6Rα, IL-11, IL-12(p40), IL-19, IL-20, IL-22, IL-26, IL-27, IL-28A, IL-29, IL-32, IL-34, IL-35, homologous to lymphotoxins, exhibits inducible expression and competes with HSV glycoprotein D for HVEM, a receptor expressed by T lymphocytes (LIGHT), matrix metalloproteinase (MMP)-1, MMP-2, MMP-3, osteocalcin, osteopontin, pentraxin-3, sTNF-R1, sTNF-R2, thymic stromal lymphopoietin (TSLP), and TNF-like weak inducer of apoptosis (TWEAK). The R&D Human Luminex Screening Assay included a disintegrin-like and metalloproteinase with thrombospondin type 1 motifs 13 (ADAMTS13), aggrecan, angiopoietin-2, B7 homolog 1/programmed death ligand 1 (B7-H1/PD-L1), bone morphogenetic protein-2 (BMP-2), sCD40, sCD40Ligand, sCD44, CX3CL1/fracktalkine, soluble E-selectin, hepatocyte growth factor (HGF), soluble intracellular adhesion molecule-1 (ICAM-1), sIFN-γR1, soluble L-selectin, leptin, leukemia inhibitory factor (LIF), oncostatin M, TNF-related apoptosis-inducing ligand (TRAIL), soluble vascular cell adhesion molecule-1 (VCAM-1), sVEGF-R2, and IL-18. The changes in serum levels of biomediators treated with tocilizumab (*n* = 11), dexamethasone (*n* = 15), or dexamethasone followed by tocilizumab (*n* = 12) were also measured using the Bio-Plex Human Cytokine 27-Plex Panel and inflammation panel. Data acquisition and analysis were performed using Bio-Plex Manager software version 5.0. The log–transformed value of all parameters measured by Multi-Plex were used for the statistical analysis.

### Quantification of Viral RNA

Viral RNA was isolated from 70 μL of serum using the QIAamp Viral RNA Mini Kit (Qiagen) according to the manufacturer's protocol and quantified using the One-Step Prime Script III RT-PCR Kit (TaKaRa) and the universal primers for N2 region of SARS-CoV-2: NIID_2019-nCOV_N_F2 AAATTTTGGGGACCAGGAAC and NIID_2019-nCOV_N_R2 TGGCAGCTGTGTAGGTCAAC with NIID_2019-nCOV_N_P2 probe: FAM-ATGTCGCGCATTGGCATGGA-TAMRA. Five microliter of the extracted RNA was used for the reaction. The PCR conditions were 25°C for 10 min for activation, 52°C for 5 min for reverse transcription, and 95°C 10 s for inactivation, followed by 45 cycles of 95°C for 5 s and 20°C for 30 s. The fluorescent signals were detected with the QuantStudio 3 Real-Time PCR System (Applied Biosystems). The amount of viral RNA in serum was successively measured in patients treated with tocilizumab (*n* = 11), dexamethasone (*n* = 15), or dexamethasone followed by tocilizumab (*n* = 12).

### Detection of the Specific Antibody Against SARS-CoV-2

The serum levels of IgG class antibody specific to SARS-CoV-2 were determined using the Corona Virus COVID-19 Antibody Rapid Detection Kit according to the manufacturer's instructions (Healgen Scientific Ltd.)^1^. Blood samples were collected from 70 patients with COVID-19 several times in each patient after 10 days of the onset of illness of the 70 patients, 10 with mild COVID-19 received only symptomatic treatment, 20 with moderate COVID-19 were treated with antiviral drugs, and 13, 15, or 12 with severe COVID-19 were treated with antiviral drug and tocilizumab (TCZ), dexamethasone (DEX), or dexamethasone followed by tocilizumab (DEX/TCZ), respectively. Induction of the IgG antibody was evaluated by two doctors blindly according to the following scale: 0 = not detectable, 1+ = faintly detectable, 2+ = between 1+ and 3+, 3+ = moderately detectable (weaker than control band), 4+ = clearly detectable (stronger than control band). In patients who showed clearly detectable in detection kit (4+), the earliest sample was plotted on the graph, while in patients who did not show clearly detectable (4+), the last sample was plotted, in order to show the maximum value and production rate of IgG antibody of each patient in each treatment group, the maximum value of antibody titer of each patient and the number of days from the onset of blood sampling date were graphed, and a logarithmic approximation curve was drawn. The maximum IgG antibody titers during the course of each patient were simply compared among the five treatment groups.

### Statistical Analysis

The significance of the difference between the well-responding group and rapidly-worsening group was evaluated using the Mann-Whitney *U*-test and a value of *P* < 0.05 was considered significant. Cytokine/chemokine values were analyzed to determine whether the raw values or log-transformed values were more normally distributed. Based on the results of this analysis, the log-transformed values were used and ANOVA was performed. For the induction of the IgG antibody against SARS-CoV-2, quantitative data (IgG antibody scales) were presented as means ± SEM and the significance of the difference between the groups was evaluated using the Mann-Whitney *U*-test with a value of *P* < 0.05 considered significant. All statistical analyses were carried out with JMP 13.0.

## Results

### Clinical Outcome of Tocilizumab Administration

The characteristics and clinical courses of patients administered with tocilizumab are summarized in Table 1. Eleven male patients and two female patients with a mean age of 63 years were evaluated. Five patients were complicated with diabetes mellitus, five with hypertension, and two with chronic obstructive pulmonary disease. At admission, two patients were critically ill and required mechanical ventilator management (MVM) before tocilizumab administration and 11 patients were diagnosed with severe disease. Tocilizumab caused no adverse events. Seven patients promptly recovered from fever and malaise, lowered their oxygen support, and were free of oxygen support within a week on average (well-responding group). PCR analysis of the nasopharyngeal specimens showed negative results for SARS-CoV-2 at 10–25 days (mean, 15.4 days) and the patients were discharged at 12–27 days (mean, 17 days) after tocilizumab administration. However, four patients showed further worsening of respiratory function and required MVM (rapidly-worsening group); these patients were administered methylprednisolone and were weaned of such a support within a week. Two patients were discharged from the hospital, and one patient died because of sudden laryngotracheal stenosis. Two patients who were critically ill and required MVM before tocilizumab injection were ameliorated in respiratory function. Analysis of clinical outcomes showed that by 1 week after the tocilizumab treatment, eight (62%), one (8%), and four (31%) patients improved, showed no change, and worsened, respectively, whereas by 1 month after the treatment, nine (69%), three (23%), and one (8%) were cured, improved, and died, respectively.

**Table 1:** Characteristics and clinical course of patients with COVID-19 treated with tocilizumab, dexamethasone or dexamethasone followed by tocilizumab.

**Case**	**Age (years)** **/Sex**	**Co-morbidities**	**Antiviral drugs**	**Required O_**2**_ concentration and laboratory findings before TCZ injection**	**Clinical course after TCZ injection**	**Outcome** **at 1 month**
**(A) TCZ monotherapy group**						
1	72/M	NTM, COPD, lower pharyngeal cancer	F+C	O_2_ 3–6 L/min CRP 8.62, Lym 175, Ferritin 153.1	Day 29: Discharge with HOT	Cure
2	41/M		F+C	O_2_ 3–5 L/min CRP 13.51, Lym 530, Ferritin 1522.6	Day 5: O_2_-free Day 13: Discharge	Cure
3	63/M	HT	F+C	O_2_ 3–5 L/min CRP 11.53, Lym 1280, Ferritin 1531.8	Day 7: O_2_-free Day 15: Discharge	Cure
4	68/M	DM	F+C	O_2_ 2–6 L/min CRP 3.93, Lym 999, Ferritin 681.1	Day 6: O_2_-free Day 19: Discharge	Cure
5	63/F	DM	F+C	O_2_ 3–6 L/min CRP 7.01, Lym 1130, Ferritin 334.3	Day 8: O_2_-free Day 18: Discharge	Cure
6	79/M	DM, HT, COPD	L/R+C	Under artificial ventilation (FiO_2_ 0.25–0.45) CRP 8.92, Lym 640, Ferritin 1238.9	Day 11: Extubation Day 14: Re-intubation Day 20: Tracheostomy O_2_ 2–3 L/min	Improvement
7	71/M	DM	L/R+C	Under artificial ventilation (FiO_2_ 0.35–0.8) CRP 7.47, Lym 2130, Ferritin 4383.4	Day 30: under artificial ventilation (FiO_2_ 0.3)	Improvement
8	79/M		F+C	O_2_ 2–5 L/min CRP 8.57, Lym 860, Ferritin 1110.7	Day 3: Intubation and transfer to other hospital, followed by mPSL pulse Day 13: Extubation and transfer to our hospital Day 15: O_2_-free Day 33: Discharge	Cure
9	48/M	DM, HT, obesity, SAS	F+C	O_2_ 5 L/min CRP 3.78, Lym 820, Ferritin 3355.4	Day 1: Intubation and transfer to other hospital, followed by mPSL administration Day 9: Extubation and transfer to our hospital Day 24: Death	Death due to sudden laryngeal stenosis
10	62/F	RA, HT, dyslipidemia	F+C	02 3–5 L/min CRP 5.06, Lym 438, Ferritin 1208.9	Day 1: Intubation and transfer to other hospital, followed by mPSL pulse Day 10: Extubation, O_2_ 5L/min Day 12: O_2_-free Day 13: Transfer to our hospital Day 25: Discharge	Cure
11	43/M	HT	F+C	O_2_ 2 L/min CRP 9.28, Lym 1129, Ferritin 552.2	Day 8: O_2_-free Day 13: Discharge	Cure
12	81/M	DM	F with mPSL	O_2_ 10–15 L/min CRP 15.26, Lym 600, Ferritin 638.6	Day 9: O_2_-free Day 22: Discharge	Cure
13	55/M		F+C	O_2_ 2–3 L/min CRP 6.77, Lym 665, Ferritin 1599.6	Day 2: Intubation Day 7: Transfer to other hospital, followed by mPSL administration Day 27: O_2_-free Day 35: Discharge	Cure
**Case**	**Age (years)** **/Sex**	**Co-morbidities**	**Antiviral drugs**	**Required O**_**2**_ **concentration and laboratory findings before DEX administration**	**Clinical course after DEX administration**	**Outcome** **at 1 month**
**(B) DEX monotherapy group**						
1	76/M	HT	F+C	O_2_ 1 L/min CRP 13.20, Lym 962, Ferritin 558.2	Day 3: O_2_-free Day 10: Discharge	Cure
2	75/F	DM, HT	F+C	O_2_ 2 L/min CRP 7.68, Lym 964, Ferritin 274.6	Day 5: O_2_-free Day 12: Discharge	Cure
3	57/M	HIV infection	F+C	O_2_ 1 L/min CRP 8.62, Lym 1810, Ferritin 327.6	Day 7: O_2_-free Day 10: Discharge	Cure
4	67/M	COPD, HT, old cerebral infarction	F+C	O_2_ 4 L/min CRP 10.62, Lym 1110, Ferritin 1235.1	Day 12: O_2_-free Day 16: Discharge	Cure
5	42/M	Nephrotic syndrome	I+C	O_2_ 1 L/min CRP 6.84, Lym 1370, Ferritin 520.7	Day 4: O_2_-free Day 10: Discharge	Cure
6	89/F	Dysphagia	F+C	O_2_ 1 L/min CRP 4.17, Lym 930, Ferritin 106.9	Day 10: O_2_-free Day 14: Discharge	Cure
7	45/F	None	F+C	O_2_ 1 L/min CRP 6.45, Lym 600, Ferritin 134.0	Day 6: O_2_-free Day 10: Discharge	Cure
8	83/M	None	None	O_2_ 1 L/min CRP 4.43, Lym 700, Ferritin 206.5	Day 4: O_2_-free Day 10: Discharge	Cure
9	78/M	COPD (HOT 1 L/min), Dysphagia	F	O_2_ 2 L/min CRP 6.53, Lym 700, Ferritin 67.5	Day 13: Discharge	Cure
10	76/M	None	F+C	02 2 L/min CRP 5.22, Lym 620, Ferritin 228.9	Day 11: O_2_-free Day 20: Discharge	Cure
11	66/M	DM COPD	F+C	O_2_ 1 L/min CRP 4.08, Lym 840, Ferritin 500.9	Day 2: O_2_ 4 L/min, followed by mPSL administration Day 15: O_2_-free Day 18: Discharge	Cure
12	80/M	Colon cancer	F+C	O_2_ 1 L/min CRP 3.75, Lym 682, Ferritin 1062.0	Day 6: O_2_-free Day 14: Complication of aspiration pneumonia Day 28: Discharge	Cure
13	66/M	DM Obesity	F+C	O_2_ 2 L/min CRP10.94, Lym 925, Ferritin 1046.2	Day 8: O_2_-free Day 14: Discharge	Cure
14	57/F	None	F+C	SpO_2_: 94% (room air) CRP 3.71, Lym 480, Ferritin 220.4	Day 11: Discharge	Cure
15	61/M	DM IHD	F+C	O_2_ 3 L/min CRP 15.47, Lym 1110, Ferritin 1197.0	Day 2: O_2_ 4 L/min, followed by mPSL administration Day 9: O_2_-free Day 16: Discharge	Cure
**Case**	**Age (years)** **/Sex**	**Co-morbidities**	**Antiviral drugs**	**Required O**_**2**_ **concentration and laboratory findings before TCZ injection (Day 1)**	**Clinical course after TCZ injection**	**Outcome** **at 1 month**
**(C) Combination therapy group of DEX and TCZ**						
1	74/F	Chronic bronchitis	F+C	Day 1: DEX and TCZ on the same day, O_2_ 3 L/min, CRP 17.78, Lym 720, Ferritin 337.3	Day 9: O_2_-free Day 18: Discharge	Cure
2	54/M	DM, HT. Obesity	F+C	Day 1: DEX and TCZ on the same day, O_2_ 3 L/min, CRP 15.50, Lym 1460, Ferritin 638.0	Day 7: O_2_-free Day 12: Discharge	Cure
3	54/M	Obesity	F+C	Day −4: DEX, O_2_ 1 L/min, CRP 2.05, Lym 890 Day 1: O_2_ 6 L/min, CRP 6.07, Lym 720, Ferritin 835.2	Day 1: Intubation and transfer to other hospital followed by mPSL administration Day 5: Extubation and transfer to our hospital, O_2_ 1 L/min Day 7: O_2_-free Day 10: Discharge	Cure
4	78/M	Chronic renal failure	F+C	Day 1: DEX and TCZ on the same day, O_2_ 3 L/min, CRP 19.33, Lym 310, Ferritin 845.3	Day 6: O_2_-free Day 11: Discharge	Cure
5	62/M	DM, Obesity	F+C	Day −1: DEX, O_2_ 1 L/min, CRP 9.11, Lym 1350, Ferritin 702.0 Day 1: O_2_ 3 L/min	Day 5: O_2_-free Day 8: Discharge	Cure
6	34/M	Obesity	F+C	Day −5: DEX, O_2_ 2 L/min, CRP 1.86, Lym 1180, Ferritin 218.2 Day 1: O_2_ 5 L/min CRP 10.88, Lym 950, Ferritin 771.8	Day 5: O_2_-free Day 8: Discharge	Cure
7	57/M	Obesity	F+C	Day −1: DEX, O_2_ 1 L/min, CRP 4.41, Lym 1090, Ferritin 268.4 Day 1: O_2_ 3 L/min, CRP 7.41, Lym 600	Day 8: O_2_-free Day 10: Discharge	Cure
8	73/M	DM, IHD	I+C	Day −6: DEX, O_2_ 2 L/min, CRP 0.94, Lym 510, Ferritin 18.3 Day 1: O_2_ 4 L/min, CRP 3.72, Lym 370	Day 9: O_2_-free Day 12: Discharge	Cure
9	79/F	DM	I+C	Day −3: DEX, O_2_ 2 L/min, CRP 1.76, Lym 560, Ferritin 382.3 Day 1: O_2_ 5 L/min	Day 5: O_2_-free Day 13: Discharge	Cure
10	59/M	DM, Obesity	F+C	Day −1: DEX, O_2_ 6 L/min, CRP 15.35, Lym 740, Ferritin 189.6 Day 1: O_2_ 10 L/min	Day 12: O_2_-free Day 17: Discharge	Cure
11	53/M	DM, Obesity, HL	F+C	Day −2: DEX, O_2_ 4 L/min, CRP 8.34, Lym 840, Ferritin 497.9 Day 1: O_2_ 5 L/min	Day 4: O_2_-free Day 16: Discharge	Cure
12	44/M	DM, Obesity	F+C	Day −2: DEX, O_2_ 3 L/min, CRP 3.17, Lym 820, Ferritin 1016.1 Day 1: O_2_ 5 L/min	Day 1: Intubation and transfer to other hospital, followed by mPSL administration Day 13: Extubation and transfer to our hospital Day 27: O_2_-free Day 29: Discharge	Cure

*TCZ, tocilizumab; NTM, non-tuberculous mycobacteriosis; COPD, chronic obstructive pulmonary diseases; DM, diabetes mellitus; HT, hypertension; IHD, ischemic heart disease; HL, hyperlipidemia; F, favipiravir; C, ciclesonide; I, ivermectin, L/R, lopinavir/ritonavir; mPSL, methylprednisolone; CRP, C-reactive protein; Lym, number of peripheral blood lymphocytes; DEX, dexamethasone*.

### Difference in Clinical Parameters Between the Well-Responding and Rapidly-Worsening Groups

**Figure 1:**
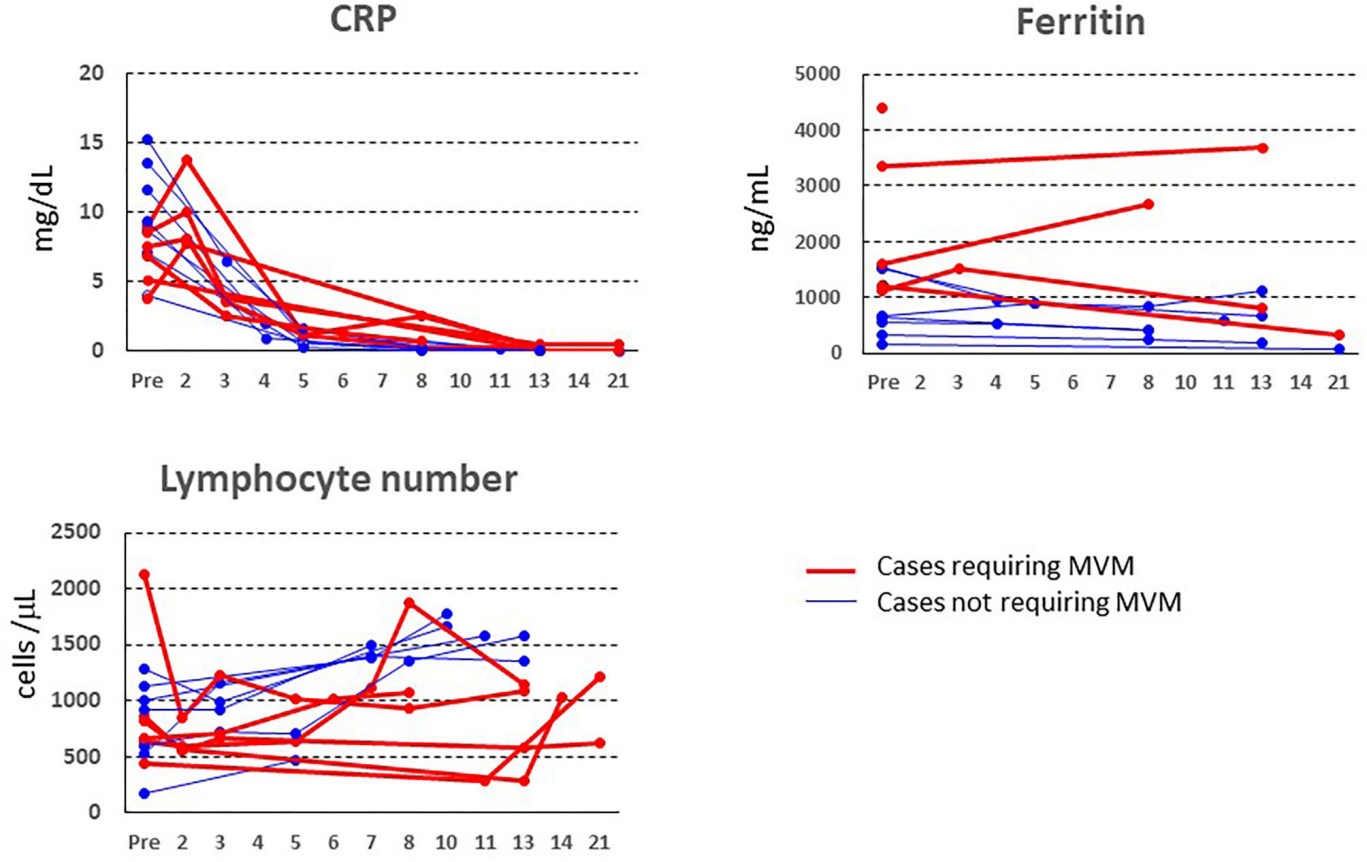
Changes in CRP, ferritin, and peripheral blood lymphocyte number before and after tocilizumab administration. The red line represents the data of patients requiring mechanical ventilation.

The changes in laboratory parameters (CRP level, ferritin level, and peripheral blood lymphocyte number) before and after tocilizumab administration are presented in Figure 1. Tocilizumab administration rapidly decreased serum CRP levels, followed by a gradual decrease in ferritin level and an increase in peripheral blood lymphocyte number; on the other hand, in three patients with the rapidly-worsening group and one critically ill patient, CRP level increased 2 days even after tocilizumab administration. In terms of the laboratory results, there were no differences in the basal levels of CRP, ferritin, or the number of peripheral blood lymphocytes between the two groups, although the ferritin:CRP ratio was significantly higher in the rapidly-worsening group than in the well-responding group (373.1 ± 346.8 vs. 83.7 ± 56.7, *P* = 0.0298; Table 2).

**Table 2:** Comparative characteristics before tocilizumab administration between the well-responding group and rapidly-worsening group.

**Characteristic**	**Well-responding group (*n* = 7)**	**Rapidly-worsening group (*n* = 4)**	***P*** **-value**
Age (years)	61.6 ± 14.7	61.0 ± 13.3	0.7763
Comorbidities	DM (*n* = 3) HT (*n* = 2) COPD (*n* = 1) NTM (*n* = 1)	DM (*n* = 1) HT (*n* = 2) Obesity (*n* = 1)	
**Laboratory data**			
CRP (mg/dL)	9.88 ± 3.88	6.05 ± 2.08	0.0726
Lymphocytes (cells/μL)	834.7 ± 404.6	730.8 ± 196.5	0.5083
Ferritin (ng/mL)	773.3 ± 546.1	1818.7 ± 1046.0	0.0726
Ferritin/CRP	83.67 ± 56.68	373.1 ± 346.8	0.0298

*DM, diabetes mellitus; HT, hypertension; COPD, chronic obstructive pulmonary diseases; NTM, non-tuberculous mycobacteriosis; CRP, C-reactive protein*.

### Serum Biomediator Levels Before and After Tocilizumab Administration

**Figure 2:**
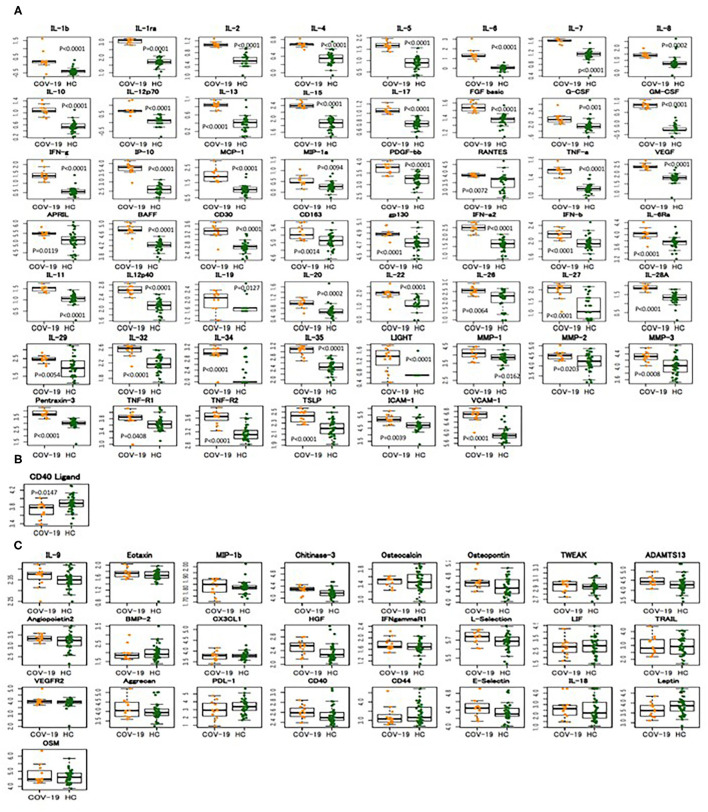
Widespread increase in biomediator levels in severely to critically ill patients with COVID-19. Expression of 80 biomediators in the sera of 12 patients with severely to critically ill patients with COVID-19 (COV-19) and 38 healthy controls (HC) was measured using a multiplex cytokine array system. The boxplots show medians (middle line) with first and third quartiles (boxes), while the whiskers show 1.5x the interquartile range (IQR) above and below the box. **(A)** Increased biomediators in COVID-19, **(B)** decreased biomediators in COVID-19, **(C)** no difference in serum levels of biomediators between COVID-19 and healthy controls.

To examine the expression of biomediators, including cytokines, chemokines, soluble receptors, and others, we measured the serum levels of 80 biomediators before and after tocilizumab administration using a multiplex cytokine array system. Compared with the healthy controls, patients with COVID-19 showed significantly elevated levels of multiple biomediators (54 out of 80, 67%) (Figure 2 and Supplementary Figure 1 and Supplementary Table 1), indicating a widespread activation of various cells in severe COVID-19. When the serum levels of 27 kinds of cytokines and chemokines were compared between patients with severe to critical COVID-19 and those with influenza, RA, or iMCD, for the latter two of which tocilizumab was approved as a therapeutic agent, it was clear that severely to critically ill patients with COVID-19 showed a broader range of high expression of these mediators (Supplementary Figure 2 and Supplementary Table 2). However, according to our results, the basal level of each cytokine did not differ between patients requiring and not requiring MVM.

**Figure 3:**
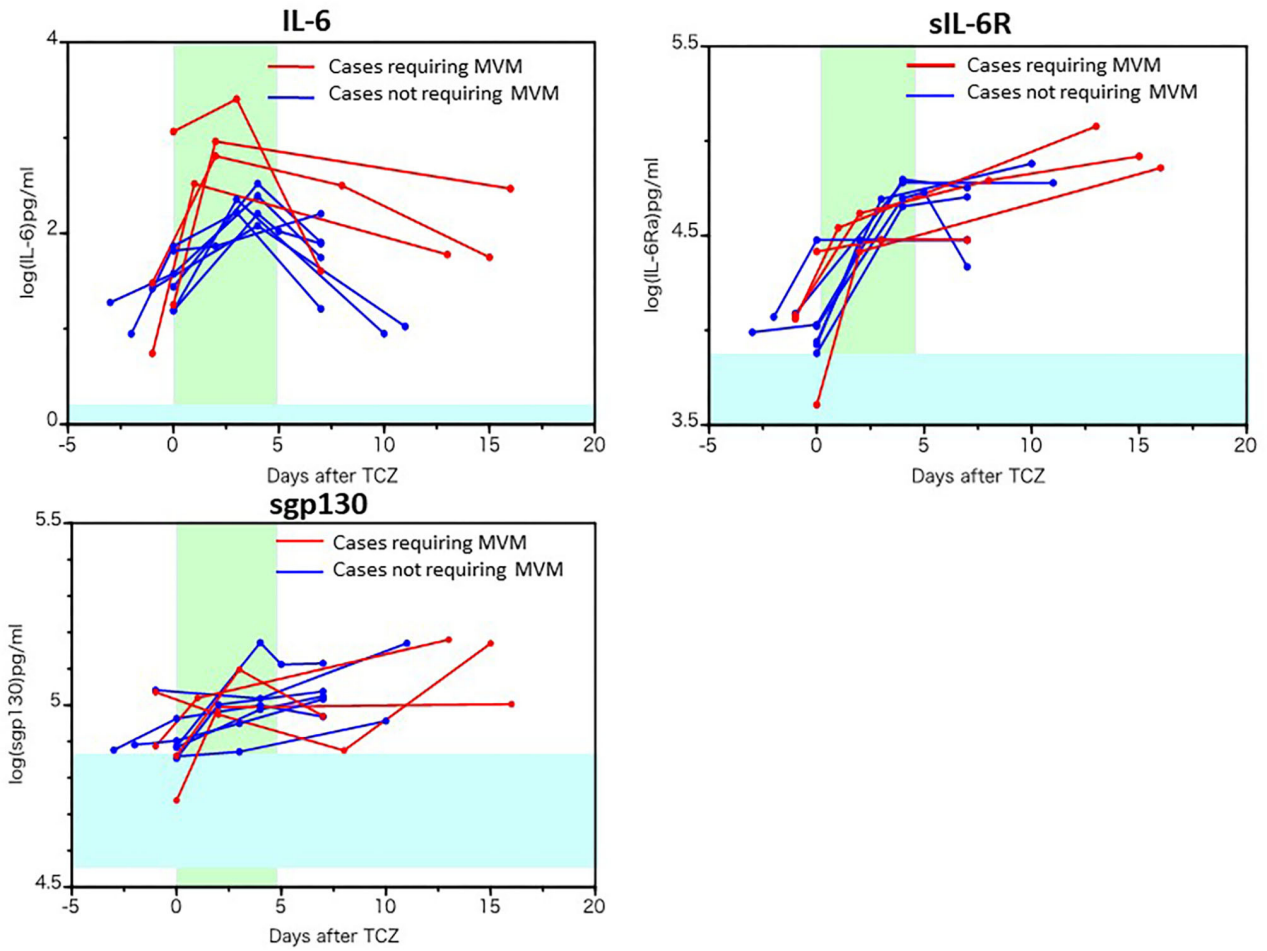
Changes in the serum levels of IL-6, soluble IL-6 receptor, and soluble gp130 before and after tocilizumab administration. The serum levels of IL-6, soluble IL-6 receptor (sIL-6R), and soluble gp130 (sgp130) in 10 patients with COVID-19 before and after tocilizumab administration were measured using a multiplex cytokine array system. The red line represents the data of patients requiring mechanical ventilation.

**Figure 4:**
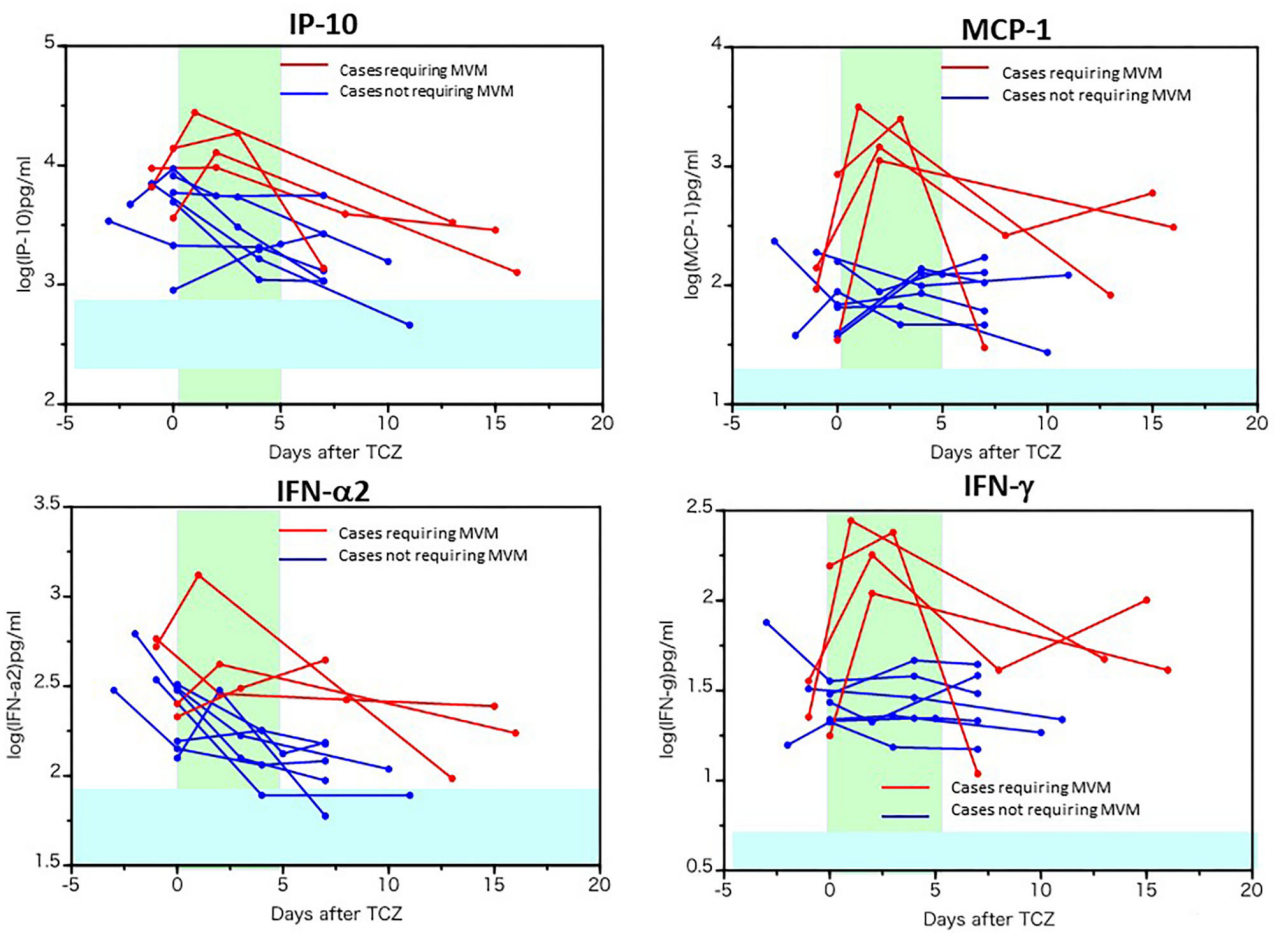
Increased cytokines and chemokines in patients requiring mechanical ventilation before and after tocilizumab administration. The serum levels of IP-10, MCP-1, IFN-α2 and γ in 10 patients with COVID-19 before and after tocilizumab injection were measured using a multiplex cytokine array system. The red line represents the data of patients requiring mechanical ventilation.

The changes in biomediator levels before and after tocilizumab administration are presented in Figures 3, 4. The levels of IL-6, sIL-6R, and soluble gp130 were further elevated after tocilizumab administration, while the level of IL-6 increased more highly and persistently in cases requiring MVM (Figure 3). Similar to that of IL-6, the levels of MCP-1 and IFN-γ were more persistently and highly elevated as well as IP-10 and IFN-α2 in cases requiring MVM than in cases not requiring MVM, implying the pathological significance of these molecules in the worsening of respiratory function or persistent activation of cells in cases requiring MVM (Figure 4). Moreover, the levels of IL-28A, soluble VCAM-1, and pentraxin-3 decreased in most patients after tocilizumab administration, while the levels of TNF-α, IL-8, IL-13, G-CSF, MIP-1α, and TWEAK tended to increase, irrespective of the clinical responsiveness to tocilizumab (Supplementary Figure 3). A single injection of tocilizumab had little effect on the serum levels of other molecules within 18 days of follow-up, as shown in Supplementary Figure 4.

### Clinical Outcome of Dexamethasone Treatment

**Figure 5:**
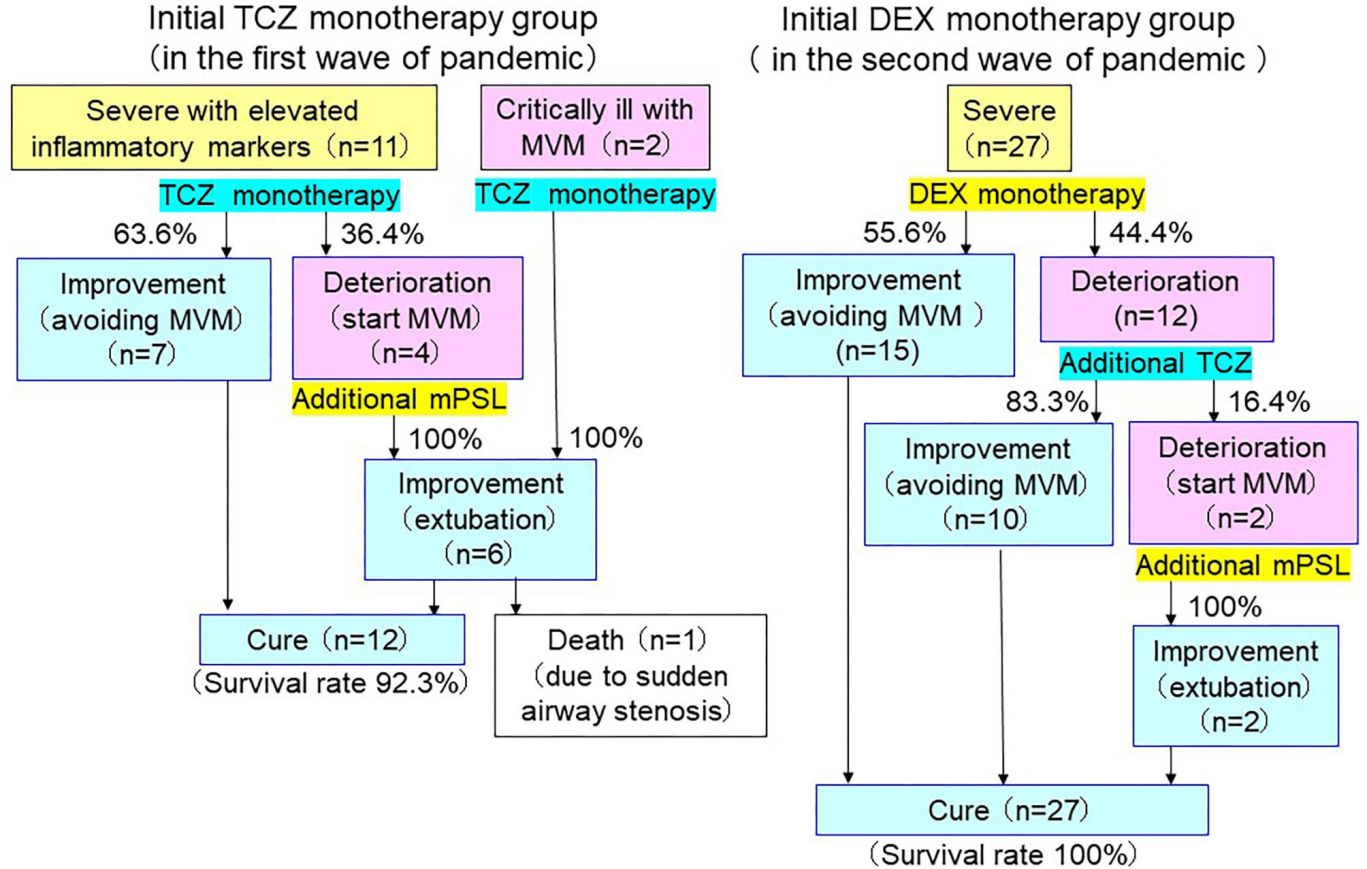
Clinical outcomes of severe-to-critically ill COVID-19. Both 7 of 11 patients (63.6%), who received tocilizumab (TCZ) monotherapy and 10 of 12 patients (83.3%), who received additional tocilizumab after rapid deterioration with dexamethasone (DEX) monotherapy were able to avoid mechanical ventilation management (MVM). Two patients, who received tocilizumab monotherapy under MVM were able to recover from MVM. However, 5 of 6 patients, who deteriorated even after single or additional tocilizumab administration and required MVM, improved with additional methylprednisolone (mPSL) treatment, and a total of 39 of 40 were cured (survival rate 97.5%).

After August, in replace of tocilizumab, we treated severe cases of COVID-19, who required oxygen supply, with dexamethasone (6 mg/day for up to 10 days), regardless of serum concentrations of CRP or ferritin. Fifteen patients responded well to dexamethasone, while 12 patients deteriorated even after corticosteroid therapy and were subsequently administered with tocilizumab. The characteristics and clinical evaluation of these patients are summarized in Figure 5 and Table 1. Among 12 patients who received the combination treatment, two patients, however, required MVM. In these patients the basal ratios of ferritin/CRP were also high (407 and 320), similarly to those of rapidly-worsening patients treated with tocilizumab. The comparison of the ratio between patients not requiring MVM and those requiring MVM is shown in Supplementary Table 3, indicating that the high ratio of ferritin/CRP is positively associated with clinical deterioration. The changes in serum levels of IL-6, IP-10, MCP-1, or IFN-γ of 15 patients treated with dexamethasone and of 12 patients treated with dexamethasone and tocilizumab are shown in Supplementary Figures 5–8. Although the dramatic increase in IL-6, IP-10, MCP-1, or IFN-γ was found in patients with COVID-19, who worsened even after tocilizumab administration, such an acute increase in these parameters were not observed after dexamethasone or dexamethasone followed by tocilizumab.

### Effects of Tocilizumab on Viral Elimination and Antibody Production

**Figure 6:**
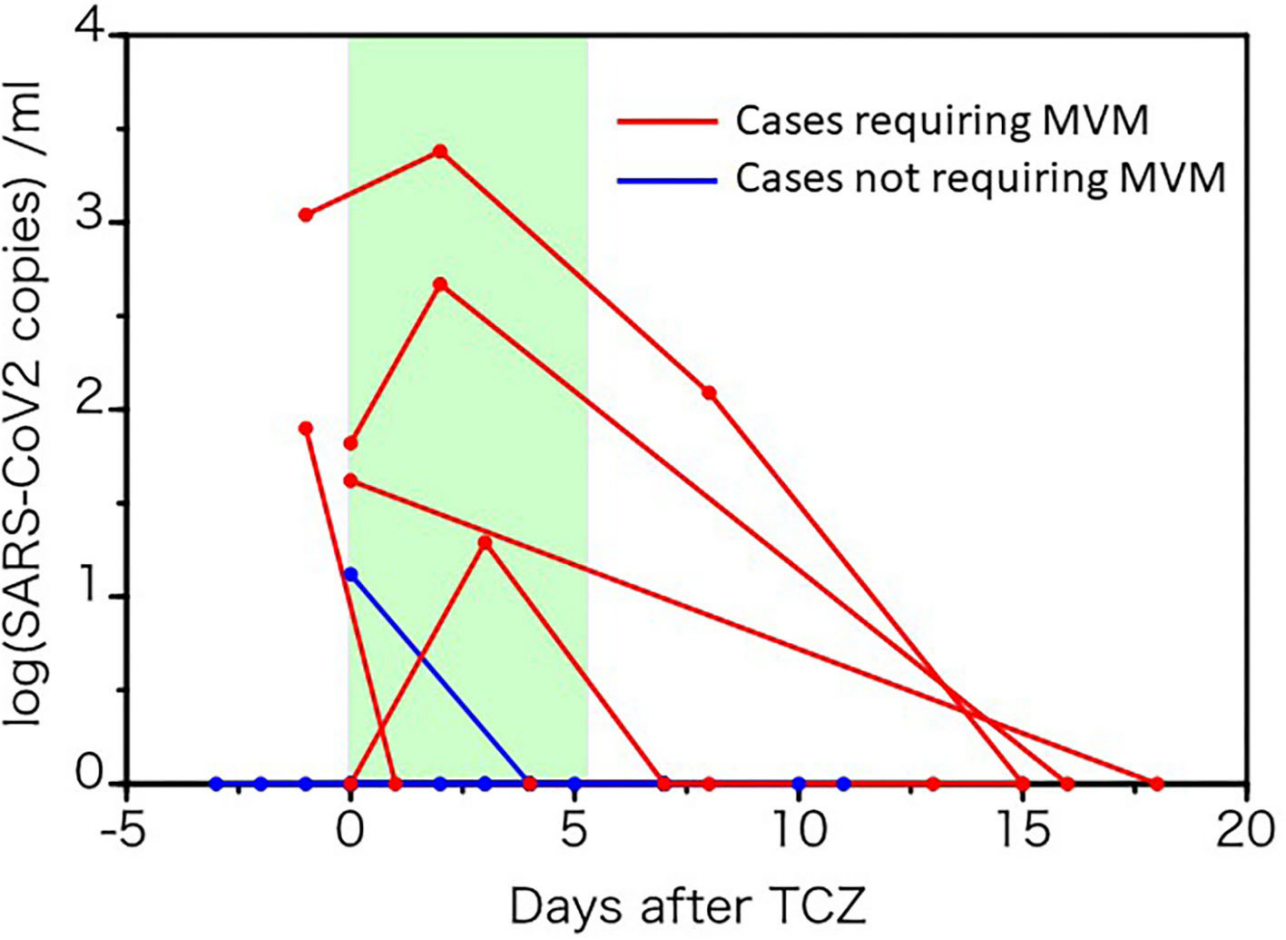
Changes in SARS-CoV-2 load before and after tocilizumab administration. Viral RNA in the sera of 10 patients with COVID-19 patients before and after tocilizumab administration were quantified by RT-PCR. The red line represents the data of patients requiring mechanical ventilation.

Because IL-6 plays a crucial role in the host defense against pathogens and promotes T-cell and B-cell activation and differentiation (22, 23), we next evaluated whether a single tocilizumab injection affects SARS-CoV-2 elimination and specific antibody production against it. Six patients, in whom five required MVM, showed viremia during the test period. Viral load transiently increased in three patients 2–3 days after tocilizumab injection, but all cases then decreased and became negative within 18 days (Figure 6). However, SARS-CoV-2 viral RNA was not detected in all 27 severely ill patients with COVID-19, who received dexamethasone with or without tocilizumab.

**Figure 7:**
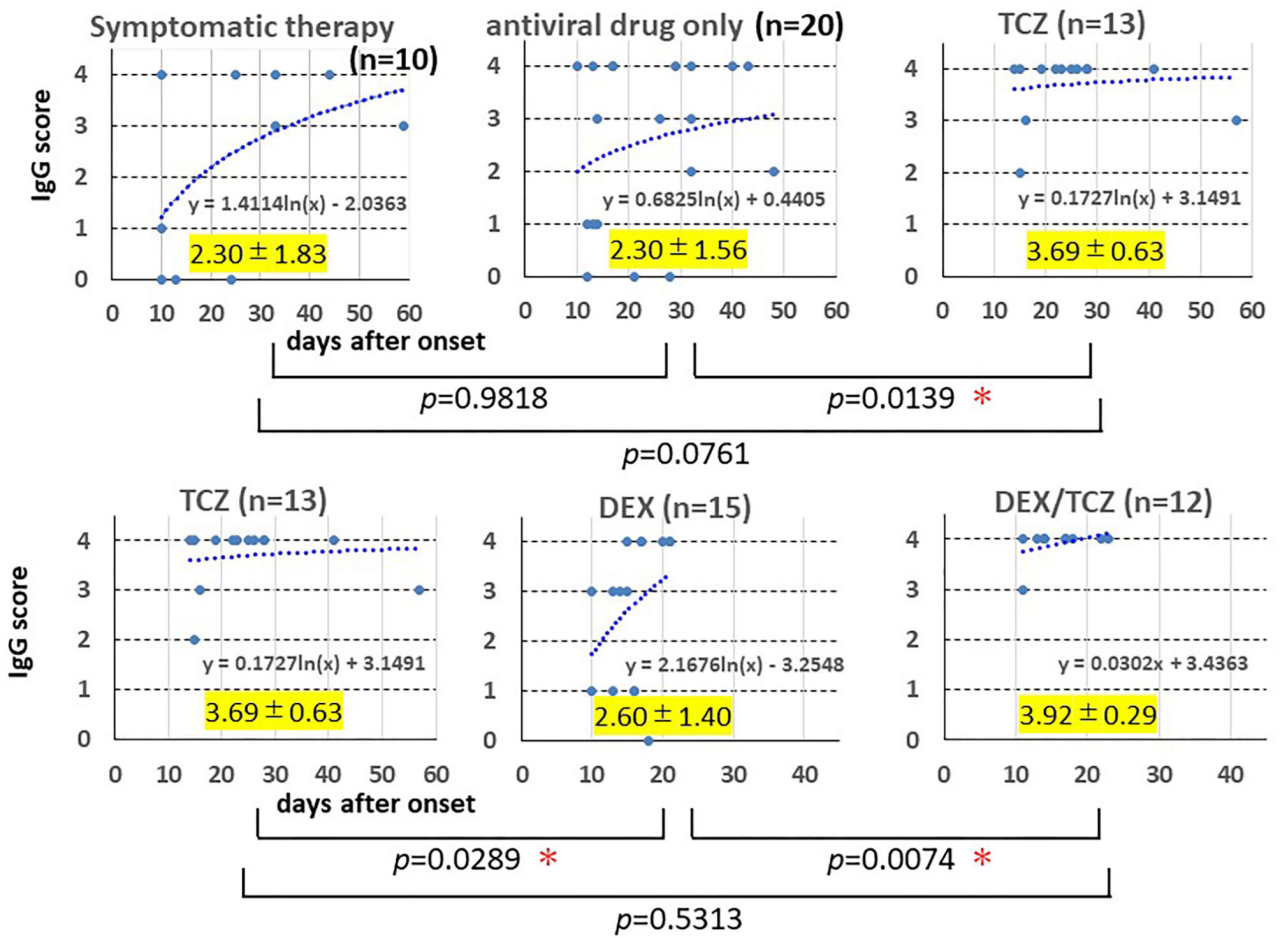
Induction of IgG antibody for SARS-CoV-2. The sera were obtained from 10 patients with mild COVID-19 receiving symptomatic therapy, 20 patients with moderate COVID-19 receiving antiviral drug(s), 13 patients with severe to critical COVID-19 receiving antiviral drug(s) plus tocilizumab, 15 patients with severe COVID-19 receiving antiviral drug(s) plus dexamethasone, and 12 patients with severe COVID-19 receiving antiviral drug(s) plus dexamethasone followed by tocilizumab. The SARS-CoV-2 specific IgG antibody titer was measured by using the Corona Virus COVID-19 Antibody Rapid Detection Kit on blood samples collected several times in each patient after 10 days of the onset of illness. In patients who showed clearly detectable in detection kit (4+), the earliest sample was plotted on the graph, while in patients who did not show clearly detectable (4+), the last sample was plotted. The horizontal axis shows the day after onset, and the vertical axis shows the antibody scale for patients in the five treatment groups. The maximum value of antibody titer of each patient and the number of days from the onset of blood sampling date were graphed, and a logarithmic approximation curve was drawn. The highest antibody titers of each patient during the course were compared among the five treatment groups. *Statistically significant.

Moreover, we evaluated and compared the maximum specific IgG antibody titers and production rate (days from onset to blood sampling date) in the course of each patient in the five groups (*n* = 70): symptomatic therapy (*n* = 10), antiviral drug only (*n* = 20), tocilizumab monotherapy (TCZ, *n* = 13), dexamethasone monotherapy (DEX, *n* = 15), dexamethasone/tocilizumab combination therapy (DEX/TCZ, *n* = 12). Figure 7 shows the result, in which the vertical axis shows the specific IgG antibody scale (maximum IgG antibody titer) and the horizontal axis shows the number of days from the onset to blood sampling (production rate of IgG antibody) for each patient in the five treatment groups. It was founded that the majority of patients who received tocilizumab monotherapy and additional tocilizumab administration produced rapid and sufficient production of specific IgG antibody, while many patients with mild-to-moderate COVID-19 (symptomatic therapy or antiviral drug only) or with severe COVID-19 treated with dexamethasone showed lower or sufficient but slow IgG antibody induction (see the logarithmic approximation curve in each treatment group), suggesting that tocilizumab in combination with antiviral drugs did not suppress synthesis of IgG class antibody specific to SARS-CoV-2, while dexamethasone suppressed antibody synthesis. A simple comparison of the maximum specific IgG antibody titers of each patient in the five treatment groups also showed significantly higher antibody titers in the tocilizumab monotherapy group and the dexamethasone/tocilizumab combination therapy group (Figure 7).

## Discussion

Several RCTs of tocilizumab for the treatment of severe to critical COVID-19 showed inconsistent results, that are likely to be due to different protocol designs, including the severity of diseases in enrolled patients, the timing of tocilizumab administration, and the concomitant use of corticosteroids (11). Recent large scale RCTs such as REMAP-CAP and RECOVERY showed the efficacy of tocilizumab in severely to critically ill patients, most of whom were also treated with corticosteroids (13, 14). However, some observational studies have demonstrated the promising efficacy of tocilizumab monotherapy in severely to critically ill patients with COVID-19 (16–19). These findings indicate that albeit not all, a substantial number of severely to critically ill patients with COVID-19 could respond to tocilizumab. Therefore, clarification of the appropriate patient population eligible for tocilizumab monotherapy or its combination with corticosteroids and the positioning of IL-6 inhibitors in COVID-19 treatment are of great importance. Here, we described our experience of administering tocilizumab to 13 severely to critically ill patients with COVID-19. Seven patients with severe disease promptly improved in response to tocilizumab (well-responding group), whereas four patients with severe disease showed worsened respiratory function and required mechanical ventilation (rapidly-worsening group). The well-responding group exhibited a comparative lower ratio of ferritin/CRP prior to tocilizumab injection and prompt reduction in serum levels of CRP, IL-6, IFN-γ, IP-10, and MCP-1 after tocilizumab administration, in comparison with the rapidly-worsening group.

Cytokine storm, a hyperinflammation state, presumably plays a major pathological role in the development of severe COVID-19 (6, 7). As shown here, the widespread overexpression of cytokines, chemokines, and soluble receptors is a characteristic of hyperinflammation in patients with severe COVID-19. Similarly, increased levels of multiple biomediators in COVID-19 have been reported (1, 24–27). Our comprehensive analysis demonstrated increased levels of a broad range of biomediators (54 out of 80) in severely to critically ill patients with COVID-19 and the expression profile of cytokines and chemokines in COVID-19 was also much broader than that in RA or iMCD, for which IL-6 blockade therapy has been already approved, suggesting activation of a wide spectrum of inflammatory cells. However, the expression profile of biomediators in COVID-19 is somewhat different from that in systemic inflammatory response syndrome (SIRS) or cytokine release syndrome (CRS) accompanied by chimeric antigen receptor (CAR)-modified T-cell therapy (22, 28). For instance, in severely or critically ill cases of SIRS or CRS, IL-6 level is dramatically elevated, usually reached values over several nanograms per milliliter. Meanwhile, IL-6 level even in severe COVID-19 is similar to (tens to hundreds of pg/mL) that in chronic inflammatory diseases such as RA or iMCD; therefore, it is questionable whether the “cytokine storm” is relevant to COVID-19 (29). The binding affinity of IL-6 to sIL-6R is ~1 nM; thus, extreme elevation of serum IL-6 level observed in severe SIRS and CRS can lead to the formation of a complex between IL-6 and sIL-6R in the serum, resulting in systemic inflammation through the activation of gp130-expressing cells, particularly vascular endothelial cells (22). Indeed, we have previously demonstrated that this complex can lead to the production of IL-6, IL-8, MCP-1, and plasminogen activator inhibitor-1 (PAI-1) by human vascular endothelial cells (28). Therefore, theoretically, the dramatic efficacy of tocilizumab in severely ill patients with CRS induced by CAR-T is mediated through suppression of the systemic activation of the trans-signaling pathway induced by the IL-6/sIL-6R complex. In COVID-19, the serum IL-6 level is far below 1 ng/mL and does not systemically stimulate vascular endothelial cells by the trans-signaling pathway; however, in the SARS-CoV-2-infected inflamed sites such as the lung or other tissues, local high IL-6 levels possibly activate the trans-signaling pathway. This may explain the difference in the effects of tocilizumab in CRS and COVID-19.

Several biomarkers such as D-dimer, CRP, ferritin, peripheral blood lymphocyte number, and neutrophil-to-lymphocyte ratio are associated with the severity and clinical outcomes in cohort studies of COVID-19, and routinely examined in hospitals (8). Patients with the rapidly-worsening group showed a significantly higher ratio of ferritin/CRP, perhaps reflecting a more severe macrophage activation. Similar effects occur in macrophage activation syndrome (MAS) complicated with systemic juvenile idiopathic arthritis (sJIA) and secondary hemophagocytic lymphohistiocytosis (6, 7), in which ferritin is highly elevated, associated with the storm of various cytokines, including IL-1, IL-2, IL-6, IL-18, IFN-γ, and others. Although the incidence of MAS in patients with sJIA appears to be lower during tocilizumab treatment, tocilizumab does not completely suppress the onset of the condition. In such cases, corticosteroids and cyclosporine are generally used. Similar to this situation, the high ratio of ferritin/CRP was closely associated with clinical deterioration in COVID-19, for which our experience suggested the requirement of the combination treatment of corticosteroids and tocilizumab, although further studies are essential to confirm this.

Overexpression of IL-6 plays a pathological role on the development of severe COVID-19 (8–10), while it is a cytokine that helps maintain homeostasis. When infections occur, IL-6 is promptly produced and plays a major role against infectious agents by producing acute phase proteins and activating T cells and B cells, leading to their differentiation into effector T cells and Ig production, respectively (22, 23). Therefore, an important concern is that tocilizumab may suppress viral elimination and humoral immunity against the virus and increase risk of opportunistic infections. It was reported that tocilizumab had increased fungal infections in patients with COVID-19 (30), whereas a living systemic review suggests that IL-6 inhibitors may not increase secondary bacterial infections (12). Our results show that one injection of tocilizumab did not suppress SARS-CoV-2 clearance and its specific IgG induction when tocilizumab is administered with a potent antiviral drug. Rather, flow cytometry of immune cells from patients with COVID-19 showed that impaired immune cell cytotoxicity in severe cases was IL-6-dependent; thus, targeting IL-6 may restore antiviral activity (31). Dexamethasone without tocilizumab appeared to delay the induction of IgG class antibody, whereas tocilizumab administration without or even with dexamethasone did not inhibit it. This observation is somewhat amazing since IL-6 plays a major role in humoral immunity. It may be due to the severity or amount of viral load. We could not detect viral load in patients treated with dexamethasone, so it remains unknown whether dexamethasone might actually affect virus elimination, however, the inhibitory unfavorable effect of dexamethasone in IgG synthesis indicates its broader suppression on defense against SARS-CoV-2 than one injection of tocilizumab.

How should hyperinflammation in COVID-19 be managed and what should be the position of IL-6 inhibitors in the treatment of severely to critically ill patients? Since the pathological characteristics of severe COVID-19 are dominated by hyperinflammation, induced by the activation of an array of cells and release of diverse biomediators, corticosteroids with broad-spectrum immune-suppressant and anti-inflammatory activity may theoretically be reasonable for treating severe COVID-19. Indeed, dexamethasone has been shown to decrease the 28-day mortality rate among patients with COVID-19 patients requiring invasive mechanical ventilation or oxygen supply (4). Thus, at present, dexamethasone is a basic immunomodulator for severely to critically ill patients with COVID-19. Of note, in RECOVERY, the combination of corticosteroids and tocilizumab improved the mortality rate compared with corticosteroids alone (12); thus, combination treatment might be considered for severe cases depending on patient characteristics. On the basis of these findings, UK Interim clinical commissioning policy and COVID-19 treatment guidelines published by National Institutes of Health USA recommended the use of tocilizumab in combination with dexamethasone in certain hospitalized patients who are exhibiting rapid respiratory decompensation or requires supplemental oxygen with a CRP level of at least 7.5 mg/dL due to COVID-19 (32, 33). More recently, WHO recommended treatment with IL-6 receptor blockers (tocilizumab or sarilumab) for patients with severe or critical COVID-19 infection (34). However, a substantial number of patients with severe COVID-19 could respond well to tocilizumab without corticosteroids. Corticosteroids, low-molecular-weight compounds, cause various adverse events, including infections, deteriorate the metabolic and atherosclerotic risk factors for severe COVID-19-related pneumonia, and thrombosis, a major cause of mortality in COVID-19. Tocilizumab administration led to significant PAI-1 suppression (28), potentially improving the hypercoagulation state. If tocilizumab monotherapy can control hyperinflammation, it would be highly advantageous. Indeed, in our cohort, the clinical characteristics and oxygen supply of 66% patients with severe COVID-19 promptly ameliorated in response to tocilizumab monotherapy without any delay of SARS-CoV-2 elimination and of induction of IgG antibody. Moreover, we found a prompt decrease in serum CRP, IL-6, IP-10, MCP-1, and IFN-γ levels in severe patients with COVID-19 who responded well to tocilizumab. An early appropriate IFN response is important for the rapid viral clearance, whereas delayed IFN response causes inflammation and tissue damage in severe COVID-19 (35). IP-10 (CXCL10) is produced by several cells in response to IFN-γ, and it acts as a chemoattractant for monocytes, macrophages, T cells, and NK cells and promotes endothelial injury (36). MCP-1 (CCL2), which is secreted by monocytes, macrophages, fibroblasts, and vascular endothelial cells via LPS or proinflammatory cytokines, attracts monocytes and basophils, and plays a pathological role on vascular damage by recruiting monocytes to the endothelial cells (36). IL-6 inhibition might ameliorate the vascular damage in patients with severe COVID-19 who did not require MVM. In addition to the reducing effect of tocilizumab on PAI-1, it is worth pointing out here that serum levels of pentraxin-3, one of vascular damage markers, were reduced in most of patients. However, patients with severe disease who required MVM did not exhibit such a prompt decrease in IL-6, IP-10, MCP-1, and IFN-γ levels. Similarly, it was reported that a prompt decrease in CRP and IL-6 was found in responders of severely ill patients with COVID-19 to tocilizumab but not in non-responders (19). These findings indicate that early reduction in CRP or these cytokines and chemokines may predict further responsiveness to tocilizumab. When these levels persistently increase, we recommend additional therapy with corticosteroids, since all four patients requiring MVM recovered with methylprednisolone administration. Alternatively, our experience suggests that patients with severe COVID-19 showing a high basal ratio of ferritin/CRP needs combinational treatment of corticosteroids and tocilizumab.

This study has several limitations. The sample size was small, and the data were analyzed retrospectively, and the treatment protocol involved a combination therapy of tocilizumab with antiviral drugs. Therefore, further studies are warranted.

## Data Availability Statement

The original contributions presented in the study are included in the article/Supplementary Material, further inquiries can be directed to the corresponding author/s.

## Ethics Statement

The studies involving human participants were reviewed and approved by Yoko Kataoka, Atsushi Ogata, Takashi Mizumori, Yoko Kondou, and Osaka Habikino Medical Center. The patients/participants provided their written informed consent to participate in this study.

## Author Contributions

SH, HK, TA, YTa, TN, and TT designed the study. SH, KY, KU, and TT wrote the manuscript. SH, KY, KU, HK, TA, YTa, HMo, HMa, YH, SM, TH, TY, YK, MK, SY, YTs, MI, EN, TS, and TN contributed to data collection and the data analysis. SH, KU, and TT prepared the figures. SH, HK, TA, YTa, HMo, HMa, YH, SM, TH, TY, YK, MK, YTs, MI, and TN contributed to clinical management of the patients. All authors contributed to the article and approved the submitted version.

## Funding

This research was supported by AMED under Grant Number 20fk0108265h0.

## Conflict of Interest

The authors declare that the research was conducted in the absence of any commercial or financial relationships that could be construed as a potential conflict of interest.

## Publisher's Note

All claims expressed in this article are solely those of the authors and do not necessarily represent those of their affiliated organizations, or those of the publisher, the editors and the reviewers. Any product that may be evaluated in this article, or claim that may be made by its manufacturer, is not guaranteed or endorsed by the publisher.

## Footnotes

^1^ https://www.healgen.com/if-respiratory-covid-19
