# Supplementary figures and images for: Prompt Reduction in CRP, IL-6, IFN-γ, IP-10, and MCP-1 and a Relatively Low Basal Ratio of Ferritin/CRP Is Possibly Associated With the Efficacy of Tocilizumab Monotherapy in Severely to Critically Ill Patients With COVID-19
Source: Front Med (Lausanne). 2021 Sep 23;8:734838. doi: 10.3389/fmed.2021.734838 (PMC8494777; doi:10.3389/fmed.2021.734838)

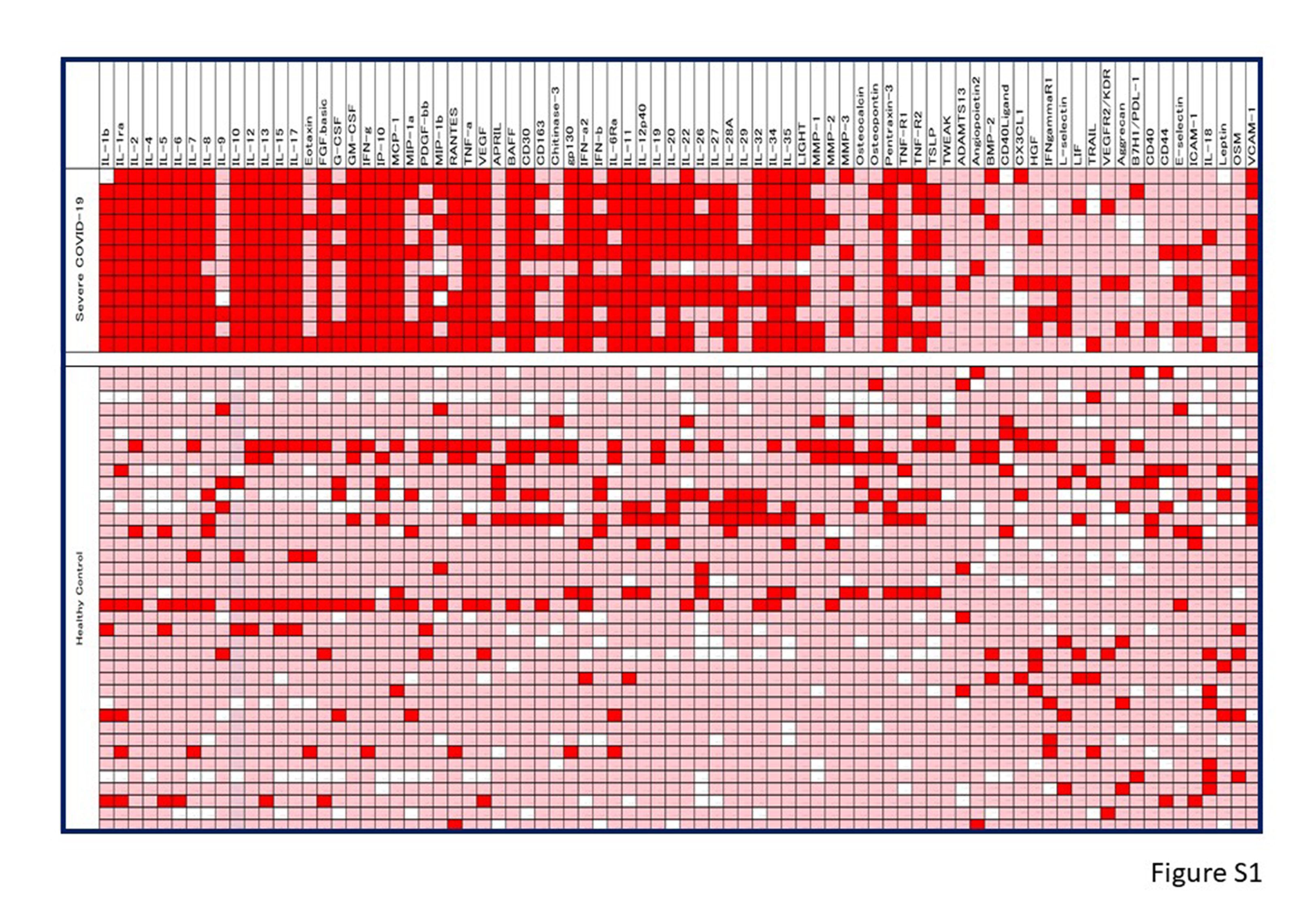

Supplement: Supplementary file 1 [file Image_1.jpg]

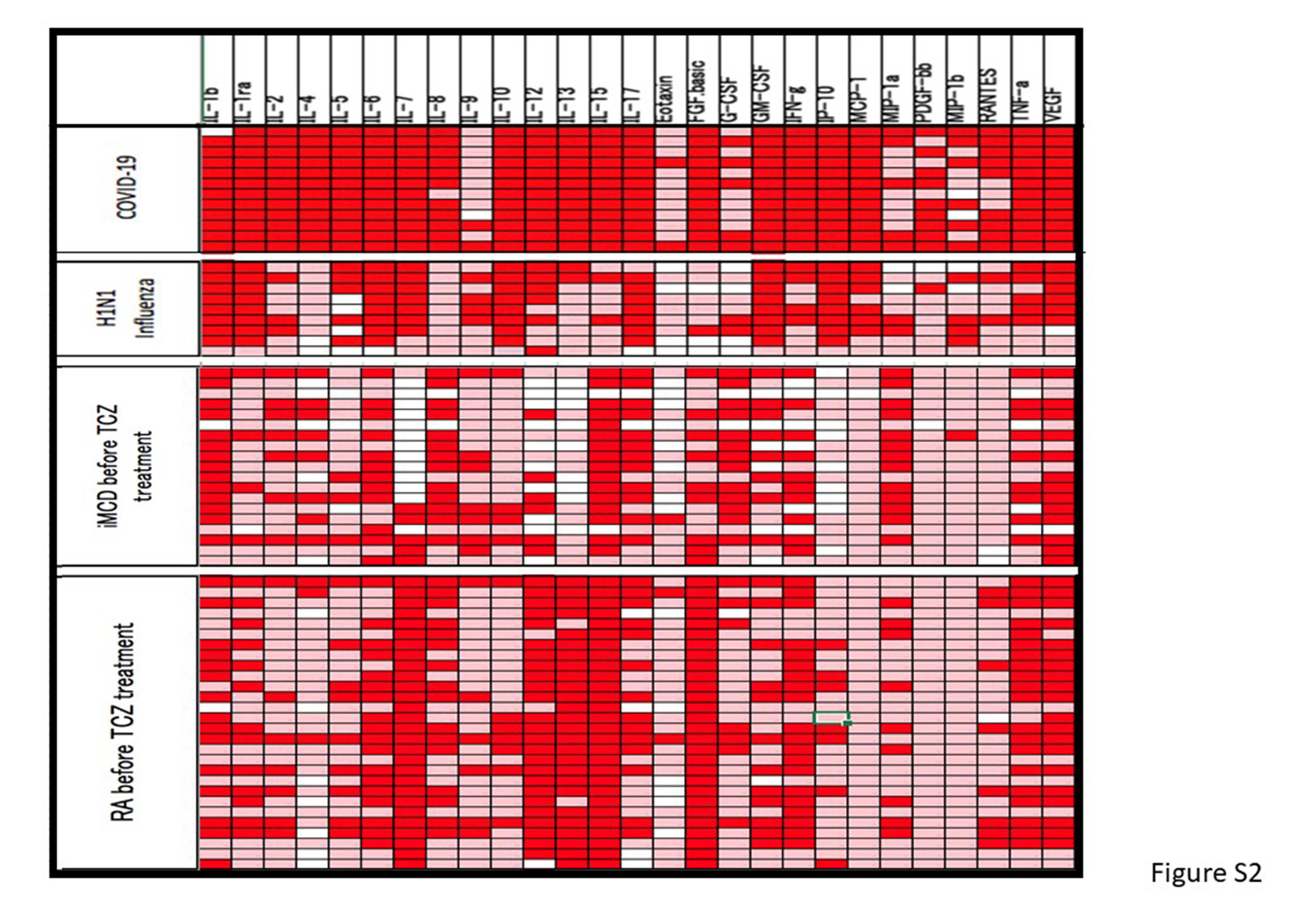

Supplement: Supplementary file 2 [file Image_2.jpg]

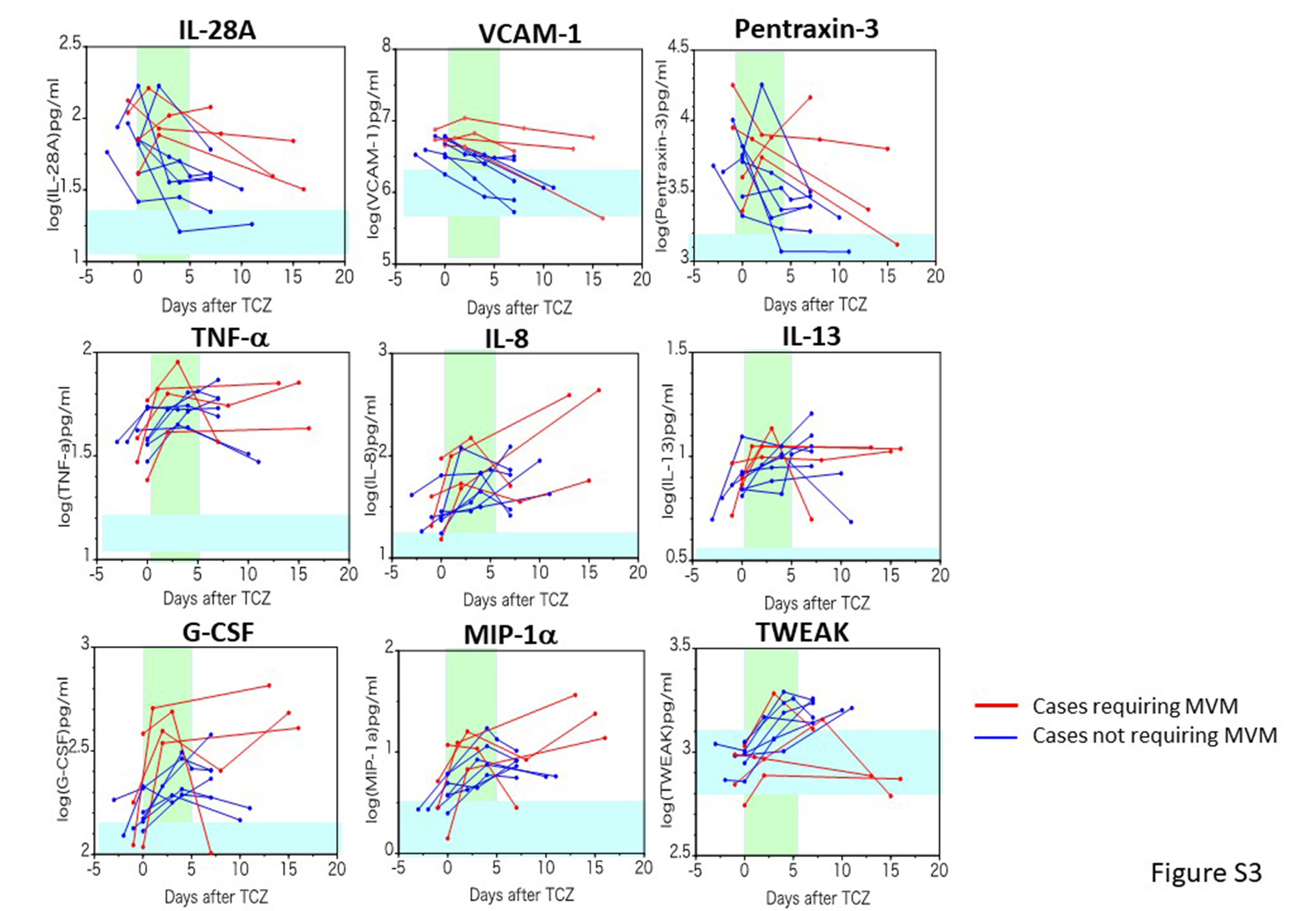

Supplement: Supplementary file 3 [file Image_3.jpg]

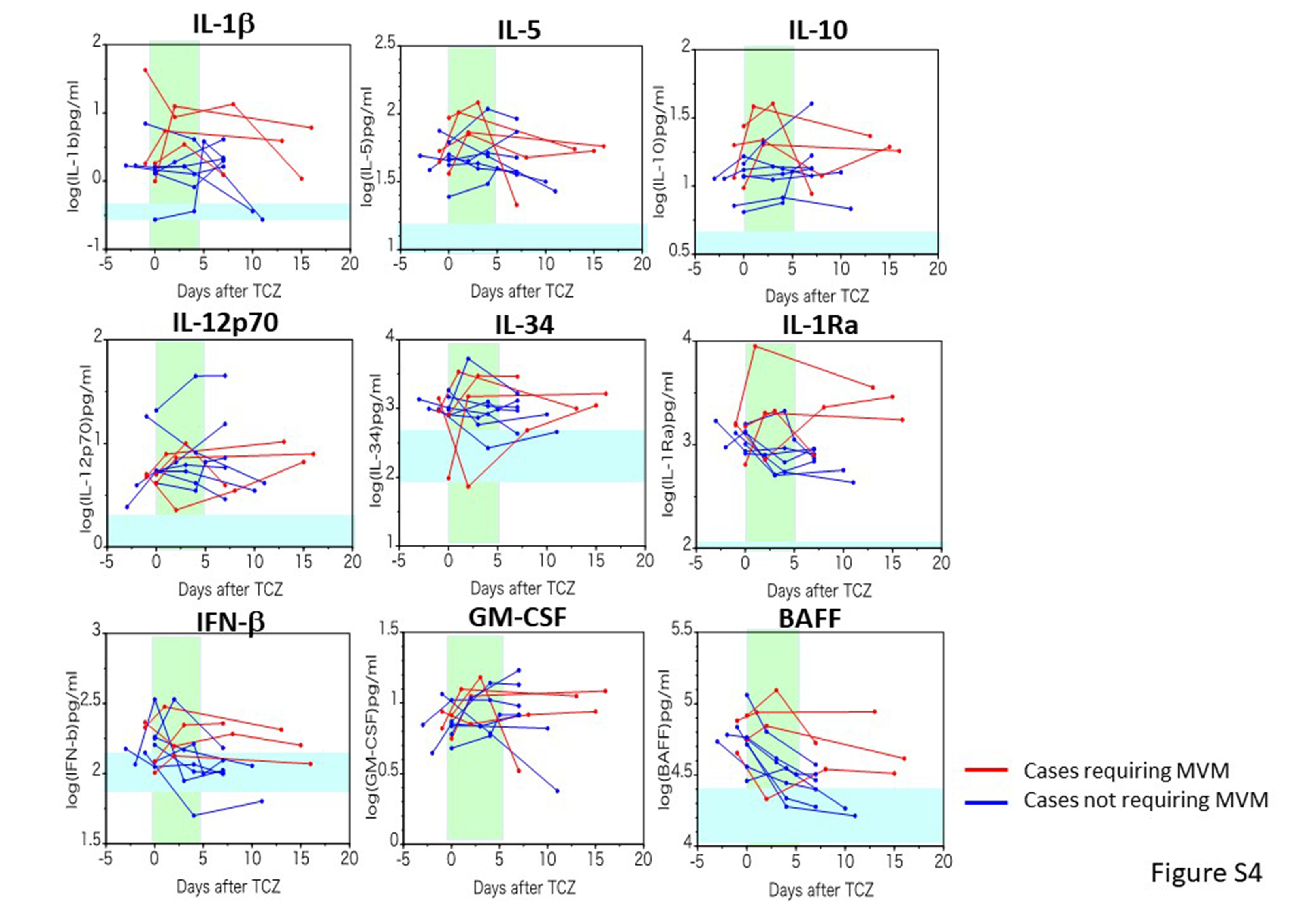

Supplement: Supplementary file 4 [file Image_4.jpg]

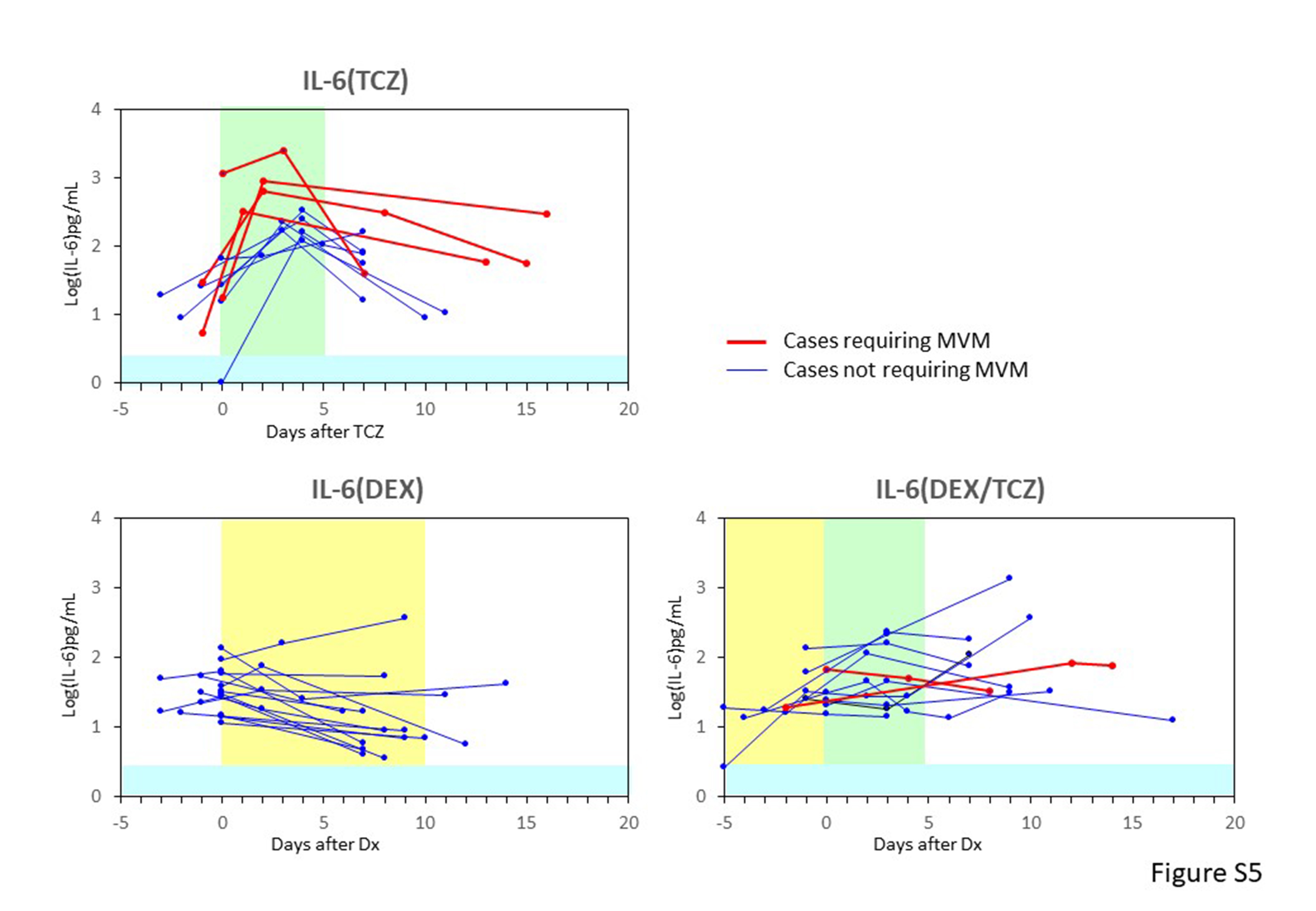

Supplement: Supplementary file 5 [file Image_5.jpg]

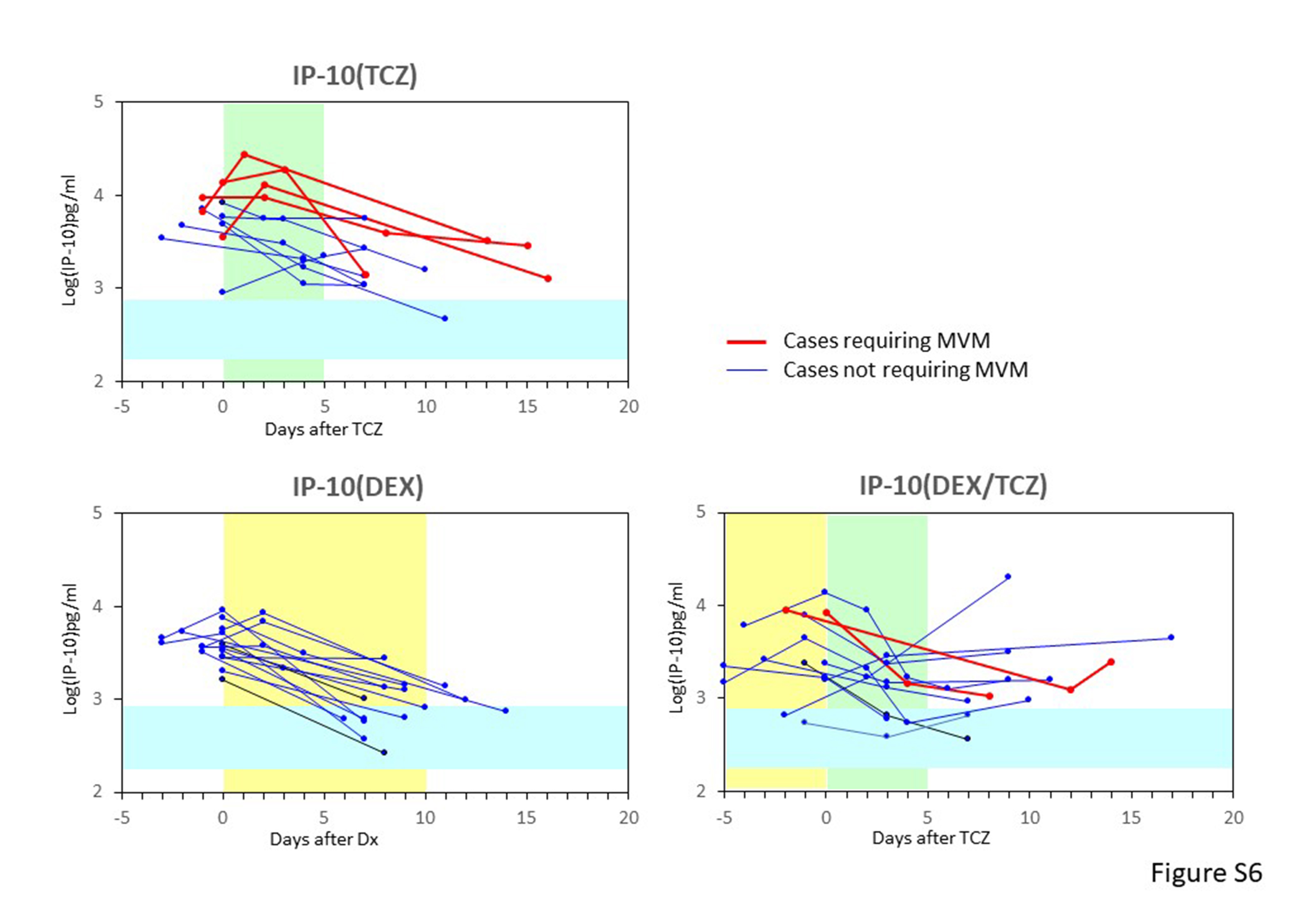

Supplement: Supplementary file 6 [file Image_6.jpg]

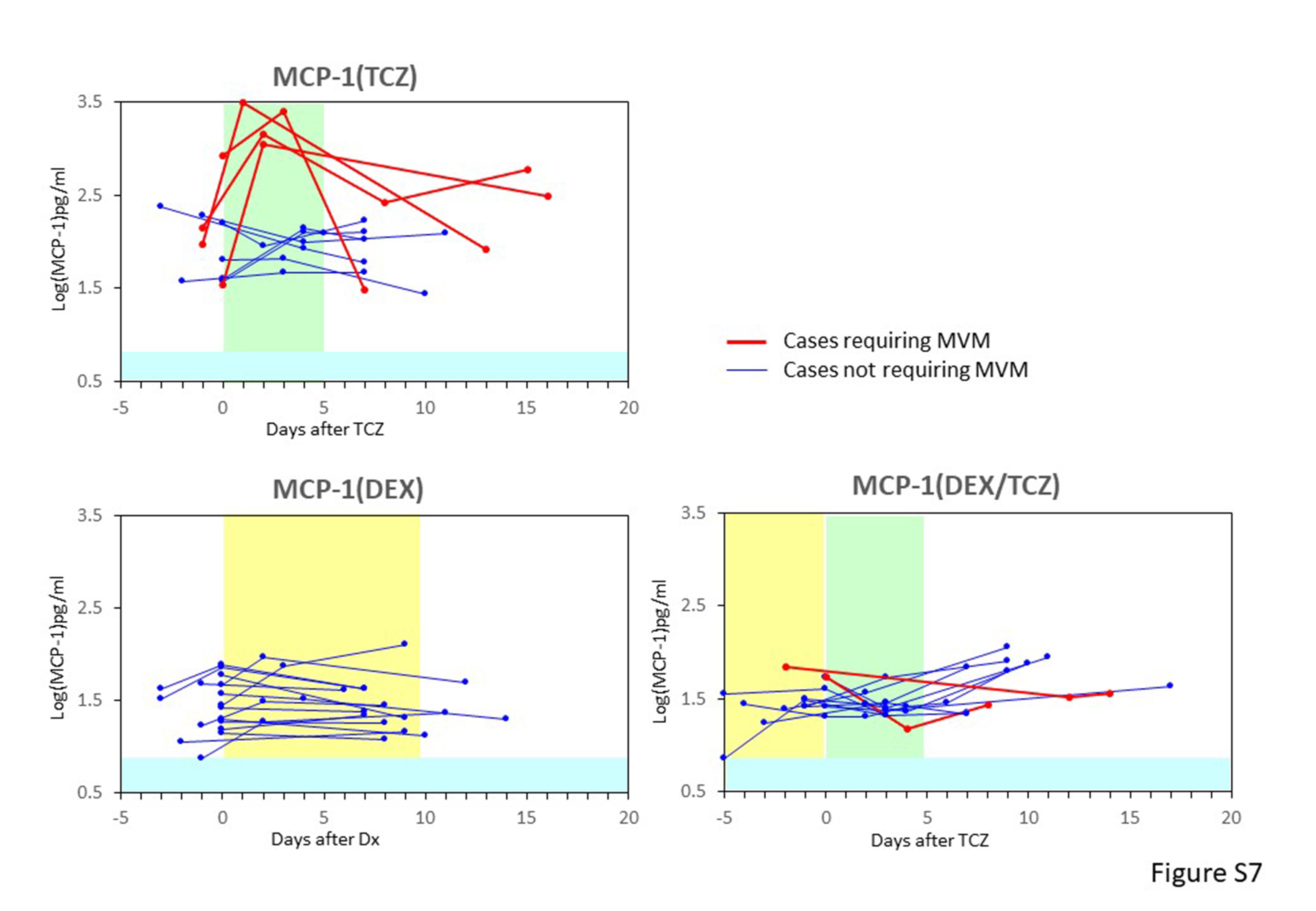

Supplement: Supplementary file 7 [file Image_7.jpg]

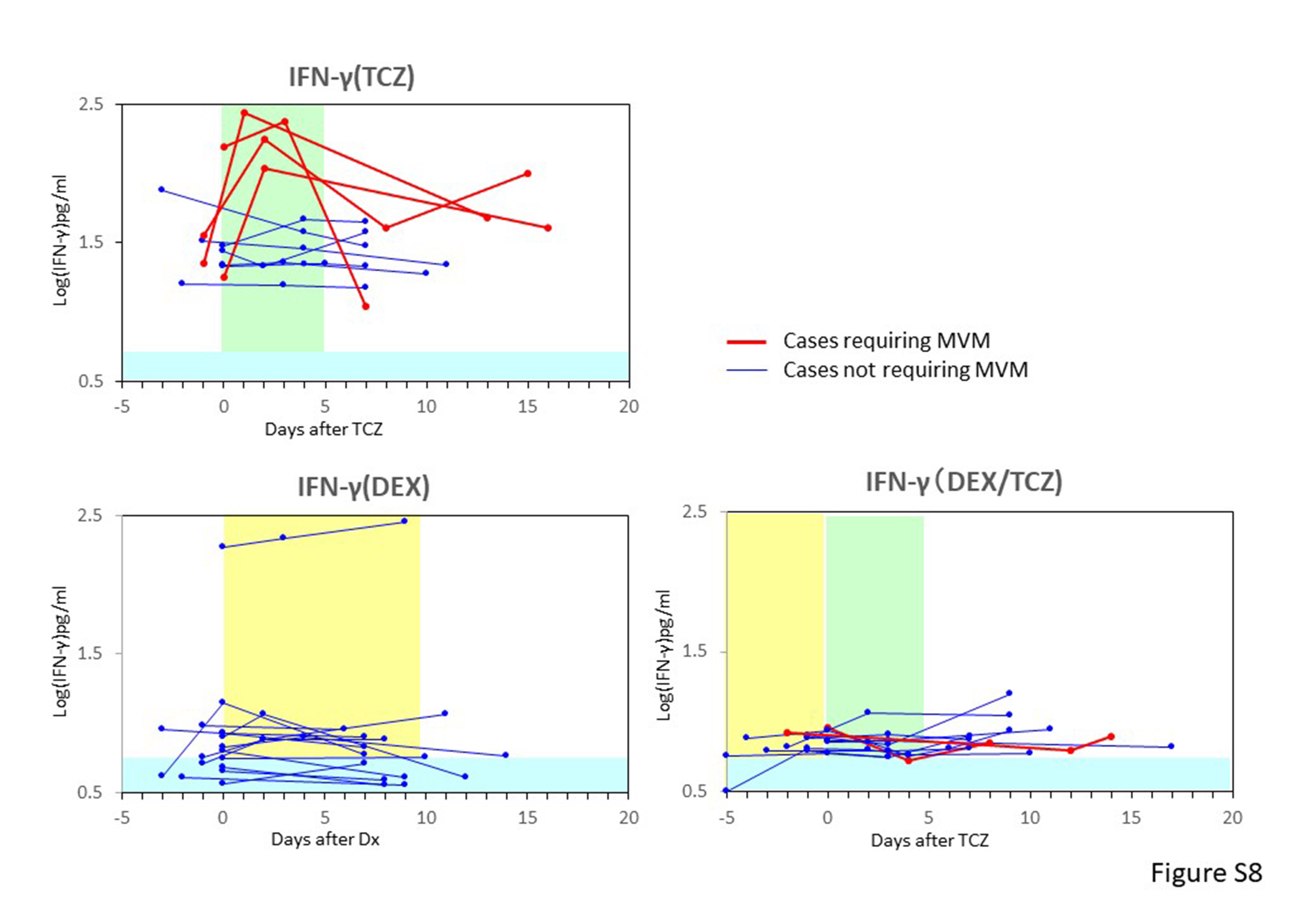

Supplement: Supplementary file 8 [file Image_8.jpg]
