# Supplementary material for: Prompt Reduction in CRP, IL-6, IFN-γ, IP-10, and MCP-1 and a Relatively Low Basal Ratio of Ferritin/CRP Is Possibly Associated With the Efficacy of Tocilizumab Monotherapy in Severely to Critically Ill Patients With COVID-19
Source: Front Med (Lausanne). 2021 Sep 23;8:734838. doi: 10.3389/fmed.2021.734838 (PMC8494777; doi:10.3389/fmed.2021.734838)
Supplement: Supplementary file 9 [file Data_Sheet_1.docx]

**Supplementary materials**

**Supplementary Figure 1. Wide-spectrum hyperactivation of biomediators in severely to critically ill patients with COVID-19 compared with that in healthy controls**

The value of each biomediator in each individual patient or healthy person is shown; the red, pink, and white boxes represent the value upper the 90% interquartile rang (IQR), within 10-90% IQR, and below 10% IQR for healthy volunteers, respectively.

**Supplementary Figure 2. Wide-spectrum hyperactivation of biomediators in severely to critically ill patients with COVID-19 compared with that in patients with influenza, rheumatoid arthritis, or idiopathic multicentric Castleman diseases**

The value of each biomediator in individual patients with COVID-19, influenza, rheumatoid arthritis (RA), or idiopathic multicentric Castleman disease (iMCD) is shown; the red, pink, and white boxes represent the values above the 90% interquartile range (IQR), within 10-90% IQR, and below 10% IQR for healthy volunteers, respectively.

**Supplementary Figure 3. Effects of tocilizumab administration on the serum levels of biomediators irrespective of its effectiveness**

The serum levels of various biomediators in 10 patients with COVID-19 before and after tocilizumab administration were measured using a multiplex cytokine array system. The red line represents the data of patients requiring mechanical ventilation.

**Supplementary Figure 4. Biomediators with negligible changes after tocilizumab administration**

The serum levels of various biomediators in 10 patients with COVID-19 before and after tocilizumab administration were measured using a multiplex cytokine array system. The red line represents the data of patients requiring mechanical ventilation support.

**Supplementary Figure 5**. **Comparison of change in the serum levels of IL-6 in patients receiving tocilizumab with those receiving dexamethasone alone or dexamethasone followed by tocilizumab**

The change in serum levels of IL-6 in 10 patients treated with tocilizumab, 15 with dexamethasone, or 12 with dexamethasone followed by tocilizumab were measured using a multiplex cytokine array system.

**Supplementary Figure 6. Comparison of change in the serum levels of IP-10 in patients receiving tocilizumab with those receiving dexamethasone alone or dexamethasone followed by tocilizumab**

The change in serum levels of IP-10 in 10 patients treated with tocilizumab, 15 with dexamethasone, or 12 with dexamethasone followed by tocilizumab were measured using a multiplex cytokine array system.

**Supplementary Figure 7. Comparison of change in the serum levels of MCP-1 in patients receiving tocilizumab with those receiving dexamethasone alone or dexamethasone followed by tocilizumab**

The change in serum levels of MCP-1 in 10 patients treated with tocilizumab, 15 with dexamethasone, or 12 with dexamethasone followed by tocilizumab were measured using a multiplex cytokine array system.

**Supplementary Figure 8. Comparison of change in the serum levels of IFN-γ in patients receiving tocilizumab with those receiving dexamethasone alone or dexamethasone followed by tocilizumab**

The change in serum levels of IFN-γ in 10 patients treated with tocilizumab, 15 with dexamethasone, or 12 with dexamethasone followed by tocilizumab were measured using a multiplex cytokine array system.

**Supplementary Table 1. Comparative analysis of 80 biomediators between severely to critically ill patients with COVID-19 and healthy volunteers**

| Biomediator | Patients with COVID-19  vs. healthy control | Patients with COVID-19 (n = 12) | | Healthy controls (n = 38) | | P value |
| --- | --- | --- | --- | --- | --- | --- |
|  |  | IQR10 | IQR90 | IQR10 | IQR90 |  |
| IL-1β | **●** | 0.0099 | 0.7848 | -0.5339 | -0.232 | <0.0001 |
| IL-1ra | **●** | 2.8191 | 3.2029 | 1.3771 | 2.0532 | <0.0001 |
| IL-2 | **●** | 1.0069 | 1.1615 | 0.3502 | 0.741 | <0.0001 |
| IL-4 | **●** | 0.62 | 0.7195 | 0.1871 | 0.5091 | <0.0001 |
| IL-5 | **●** | 1.5084 | 1.8664 | 0.4872 | 1.1645 | <0.0001 |
| IL-6 | **●** | 0.9723 | 1.784 | 0.1667 | 0.3152 | <0.0001 |
| IL-7 | **●** | 1.4866 | 1.6735 | 1.0153 | 1.2889 | <0.0001 |
| IL-8 | **●** | 1.2429 | 1.6142 | 0.5152 | 1.2177 | 0.0002 |
| IL-10 | **●** | 0.8686 | 1.2876 | 0.3688 | 0.6839 | <0.0001 |
| IL-12p70 | **●** | 0.5966 | 1.207 | 0.2575 | 0.3616 | <0.0001 |
| IL-13 | **●** | 0.7247 | 0.9102 | 0.2068 | 0.5439 | <0.0001 |
| IL-15 | **●** | 2.3266 | 2.5021 | 1.6319 | 2.0233 | <0.0001 |
| IL-17 | **●** | 1.0035 | 1.1481 | 0.6542 | 0.9059 | <0.0001 |
| FGF basic | **●** | 1.4691 | 1.6142 | 1.2964 | 1.4268 | <0.0001 |
| G-CSF | **○** | 2.0367 | 2.2622 | 1.8213 | 2.1586 | 0.001 |
| GM-CSF | **●** | 0.6489 | 0.9365 | 0.3010 | -0.005 | <0.0001 |
| IFN-γ | **●** | 1.2025 | 1.8459 | 0.3909 | 0.7251 | <0.0001 |
| IP-10 | **●** | 3.5362 | 3.9697 | 2.3603 | 2.9077 | <0.0001 |
| MCP-1 | **●** | 1.5704 | 2.3613 | 0.7735 | 1.2747 | <0.0001 |
| MIP-1α | **○** | 0.396 | 0.7773 | 0.0569 | 0.5146 | 0.0094 |
| PDGF-bb | **○** | 3.3919 | 3.884 | 2.8752 | 3.5481 | <0.0001 |
| RANTES | **○** | 3.8276 | 4.0181 | 3.0038 | 3.8616 | 0.0072 |
| TNF-α | **●** | 1.4717 | 1.6204 | 1.0643 | 1.2473 | <0.0001 |
| VEGF | **●** | 2.2713 | 2.5472 | 1.6348 | 1.9815 | <0.0001 |
| APRIL | **○** | 5.3767 | 5.5686 | 4.3750 | 5.7609 | 0.0119 |
| BAFF | **●** | 4.6606 | 5.003 | 4.0075 | 4.4101 | <0.0001 |
| CD30 | **●** | 3.1648 | 3.5269 | 2.5033 | 2.9448 | <0.0001 |
| CD163 | **○** | 5.0766 | 5.5176 | 4.7934 | 5.2513 | 0.0014 |
| gp130 | **●** | 4.8543 | 5.0248 | 4.5782 | 4.8446 | <0.0001 |
| IFN-α2 | **●** | 2.2076 | 2.7604 | 1.1613 | 1.9158 | <0.0001 |
| IFN-β | **○** | 2.0674 | 2.3218 | 1.7856 | 2.1596 | <0.0001 |
| IL-6R | **○** | 3.8835 | 4.22 | 3.5604 | 3.9087 | <0.0001 |
| IL-11 | **●** | 1.3685 | 1.5861 | 0.8315 | 1.2517 | <0.0001 |
| IL-12p40 | **●** | 2.485 | 2.762 | 1.7416 | 2.3214 | <0.0001 |
| IL-19 | **○** | 1.2808 | 2.3356 | 1.5775 | 1.699 | 0.0127 |
| IL-20 | **○** | 0.8145 | 1.1593 | 0.5259 | 0.8431 | 0.0002 |
| IL-22 | **○** | 1.7074 | 2.1473 | 0.9917 | 1.7246 | <0.0001 |
| IL-26 | **○** | 2.3395 | 2.7494 | 1.3646 | 2.6079 | 0.0064 |
| IL-27 | **○** | 1.6389 | 2.1982 | 0.6990 | 1.9372 | <0.0001 |
| IL-28A | **●** | 1.6301 | 2.034 | 1.0726 | 1.6263 | <0.0001 |
| IL-29 | **○** | 2.2233 | 2.6543 | 1.4082 | 2.6303 | 0.0054 |
| IL-32 | **○** | 2.3895 | 2.6488 | 1.9209 | 2.3912 | <0.0001 |
| IL-34 | **●** | 2.9013 | 3.1675 | 1.9856 | 2.7424 | <0.0001 |
| IL-35 | **●** | 2.785 | 3.1905 | 2.1587 | 2.7488 | <0.0001 |
| LIGHT | **●** | 0.7131 | 1.7554 | 0.6990 | 0.699 | <0.0001 |
| MMP-1 | **○** | 3.6236 | 4.4501 | 3.4887 | 4.046 | 0.0162 |
| MMP-2 | **○** | 4.2617 | 4.6577 | 3.7265 | 4.7071 | 0.0203 |
| MMP-3 | **○** | 4.1476 | 4.4376 | 3.6502 | 4.3584 | 0.0008 |
| Pentraxin-3 | **●** | 3.474 | 4.0032 | 2.7441 | 3.2987 | <0.0001 |
| TNF-R1 | **○** | 3.7311 | 3.999 | 3.4325 | 3.8955 | 0.0408 |
| TNF-R2 | **○** | 3.3826 | 3.7693 | 2.9591 | 3.4535 | <0.0001 |
| TSLP | **○** | 2.2892 | 2.5139 | 2.0528 | 2.3807 | <0.0001 |
| ICAM-1 | **○** | 4.7919 | 5.4964 | 4.3468 | 5.2878 | 0.0039 |
| VCAM-1 | **●** | 6.4939 | 6.8669 | 5.6460 | 6.2747 | <0.0001 |
| CD40 ligand | ▽ | 3.4805 | 3.8732 | 3.6775 | 4.0996 | 0.0147 |
| IL-9 | **−** | 2.3162 | 2.4014 | 2.3063 | 2.4013 | 0.287 |
| Eotaxin | **−** | 1.5956 | 1.9346 | 1.5182 | 1.8486 | 0.1973 |
| MIP-1β | **−** | 1.7354 | 1.8992 | 1.7653 | 1.8594 | 0.7347 |
| Chitinase-3 | **−** | 4.0827 | 4.3974 | 4.0191 | 4.3763 | 0.2272 |
| Osteocalcin | **−** | 3.3123 | 3.5788 | 3.2334 | 3.8222 | 0.943 |
| Osteopontin | **−** | 4.3493 | 4.9883 | 4.1993 | 4.8512 | 0.0881 |
| TWEAK | **−** | 2.8444 | 3.0448 | 2.8047 | 3.1177 | 0.9904 |
| ADAMTS13 | **−** | 4.2802 | 4.6612 | 3.8640 | 4.7995 | 0.2583 |
| Angiopoietin 2 | **−** | 3.2068 | 3.5471 | 2.6855 | 3.5434 | 0.2049 |
| BMP-2 | **−** | 1.6517 | 2.5195 | 1.6060 | 2.4353 | 0.8453 |
| CX3CL1 | **−** | 3.5621 | 3.9967 | 3.5809 | 4.0041 | 0.6054 |
| HGF | **−** | 2.2118 | 2.7832 | 2.1244 | 2.585 | 0.0509 |
| IFN-γR1 | **−** | 1.6211 | 2.0569 | 1.5253 | 2.0136 | 0.4265 |
| L-selectin | **−** | 5.6182 | 5.8125 | 5.5690 | 5.7758 | 0.0712 |
| LIF | **−** | 2.0245 | 3.2535 | 1.8406 | 3.6561 | 0.8191 |
| TRAIL | **−** | 2.2144 | 3.6205 | 2.2583 | 3.8323 | 0.969 |
| VEGFR2/KDR | **−** | 3.8306 | 4.1547 | 3.6929 | 4.1184 | 0.1913 |
| Aggrecan | **−** | 3.6931 | 4.5648 | 3.6181 | 4.5275 | 0.3012 |
| B7H1/PDL-1 | **−** | 1.6963 | 4.2873 | 2.2280 | 4.3619 | 0.1788 |
| CD40 | **−** | 2.4965 | 2.7348 | 2.3678 | 2.7772 | 0.2566 |
| CD44 | **−** | 2.8498 | 3.7125 | 2.8571 | 4.0516 | 0.4728 |
| E-selectin | **−** | 4.1959 | 4.6238 | 4.0900 | 4.5373 | 0.1243 |
| IL-18 | **−** | 1.6117 | 4.2564 | 1.4655 | 4.3979 | 0.1591 |
| Leptin | **−** | 3.2791 | 4.0401 | 3.3168 | 4.3913 | 0.1714 |
| OSM | **−** | 4.3158 | 5.451 | 4.1433 | 5.0832 | 0.3768 |

Elevated beyond the control range (**●**), 32 (40%)

Significantly elevated beyond the control range (**○**), 22 (27.5%)

Healthy control range (−), 25 (31.25%)

Significantly decreased below the control range (▽), 1 (1.25%)

Total biomolecules, 80

**Supplementary Table 2. Comparative analysis of 27 biomediators between patients with severe to critical COVID-19, influenza, rheumatoid arthritis, or idiopathic multicentric Castleman disease and healthy volunteers**

| Biomediator | Patients with COVID-19 vs. Healthy controls | | Patients with Influenza vs. Healthy controls | | Patients with RA vs.  Healthy controls | | Patients with iMCD vs.  Healthy controls | |
| --- | --- | --- | --- | --- | --- | --- | --- | --- |
|  |  | P value |  | P value |  | P value |  | P value |
| IL-1β | ● | <0.0001 | ○ | 0.0433 | ○ | 0.0026 | ○ | 0.0008 |
| IL-1ra | ● | <0.0001 | ○ | 0.0007 | ○ | <0.0001 | − |  |
| IL-2 | ● | <0.0001 | − |  | ○ | 0.0003 | ○ | 0.0003 |
| IL-4 | ● | <0.0001 | − |  | − |  | − |  |
| IL-5 | ● | <0.0001 | ○ | 0.0371 | ○ | 0.0195 | − |  |
| IL-6 | ● | <0.0001 | ● | <0.0001 | ○ | <0.0001 | ○ | <0.0001 |
| IL-7 | ● | <0.0001 | − |  | ● | <0.0001 | − |  |
| IL-8 | ● | 0.0002 | − |  | ○ | 0.0033 | ○ | <0.0001 |
| IL-9 | − |  | − |  | − |  | ○ | 0.0355 |
| IL-10 | ● | <0.0001 | ● | <0.0001 | − |  | − |  |
| IL-12p70 | ● | <0.0001 | ○ | 0.0303 | ● | <0.0001 | − | 0.683 |
| IL-13 | ● | <0.0001 | − |  | ● | <0.0001 | ▽ | 0.0014 |
| IL-15 | ● | <0.0001 | − |  | ● | <0.0001 | ○ | <0.0001 |
| IL-17 | ● | <0.0001 | − | 0.0589 | ○ | 0.0208 | ○ | <0.0001 |
| Eotaxin | − |  | − |  | − |  | ▽ | 0.0085 |
| FGF basic | ● | <0.0001 | − |  | ● | <0.0001 | ○ | 0.0193 |
| G-CSF | ○ | 0.001 | ○ | 0.0057 | − |  | ○ | <0.0001 |
| GM-CSF | ● | <0.0001 | − |  | ○ | 0.0012 | − |  |
| IFN-γ | ● | <0.0001 | − |  | ○ | <0.0001 | − |  |
| IP-10 | ● | <0.0001 | ● | <0.0001 | ○ | 0.0004 | ▽ | 0.0001 |
| MCP-1 | ● | <0.0001 | ● | 0.0005 | − |  | − |  |
| MIP-1α | ○ | 0.0094 | ○ | 0.0464 | ○ | 0.0388 | ○ | <0.0001 |
| PDGF-bb | ○ | <0.0001 | ▽ | 0.0327 | − |  | − |  |
| MIP-1β | − |  | − |  | − |  | − | 0.0612 |
| RANTES | ○ | 0.0072 | ● | 0.0027 | ○ | 0.005 | − |  |
| TNF-α | ● | <0.0001 | − | 0.0752 | ○ | <0.0001 | − | 0.0603 |
| VEGF | ● | <0.0001 | − |  | ○ | <0.0001 | ○ | 0.0001 |

|  | Numbers of cytokines or chemokines (%) | | | |
| --- | --- | --- | --- | --- |
| Healthy controls vs. | COVID-19 | Influenza | RA | iMCD |
| Elevation beyond the control range (●) | 20 (74.1%) | 5 (18.5%) | 5 (18.5%) | 0 |
| Significantly elevated beyond the control range (○） | 4 (14.8%) | 6 (22.2%) | 14 (51.9%) | 12 (44.4%) |
| Healthy control range (−) | 3 (11.1%) | 15 (55.6%) | 8 (29.6%) | 12 (44.4%) |
| Significantly decreased below the control range (▽) | 0 | 1 (3.7%) | 0 | 3 (11.1%) |
| Total | 27 | 27 | 27 | 27 |

**Supplementary Table 3. A high Ferritin/CRP ratio is associated with the development of severe COVID-19 into mechanical ventilation support**

|  | Recovery without MV support (n = 32) | Development into MV support (n = 6) | P value |
| --- | --- | --- | --- |
| Age (years) | 64.8±13.8 | 57.0±12.4 | 0.186 |
| Treatment  TCZ monotherapy  DEX monotherapy  DEX → TCZ | 7  15  10 | 4 (followed by mPSL)  0  2 (followed by mPSL) |  |
| Laboratory data  CRP (mg/dL)  Ferritin (ng/mL)  Ferritin/CRP | 8.98±4.77  555.0±415.2  68.1±50.3 | 5.57±1.99  1521.0±934.0  325.1±284.7 | 0.0685  0.0025  0.0004 |

TCZ, tocilizumab; DEX, dexamethasone; MV, mechanical ventilation; CRP, C-reactive protein.
